# Prescriptions patterns and appropriateness of usage of antibiotics in non-teaching community hospitals in South Korea: a multicentre retrospective study

**DOI:** 10.1186/s13756-022-01082-2

**Published:** 2022-02-22

**Authors:** Yong Chan Kim, Ji Young Park, Bongyoung Kim, Eu Suk Kim, Hyuk Ga, Rangmi Myung, Se Yoon Park, Myung Jin Lee, Song Mi Moon, Sun Hee Park, Kyoung-Ho Song, Hong Bin Kim, Korea Study Group for Antimicrobial Stewardship (KOSGAP), Jinwoo Yang, Hyunok Park, Ji Hoon Kang, Myoungsuk Yun, Young Taek Kim, Hye Jung Lee, Woochang Hwang

**Affiliations:** 1 Department of Internal Medicine, Division of Infectious Diseases, Yongin Severance Hospital, Yonsei University College of Medicine, Yongin, Korea; 2 Department of Pediatrics, Chung-Ang University Hospital, Seoul, Korea; 3 Department of Internal Medicine, Hanyang University College of Medicine, 222-1, Wangsimni-ro, Seongdong-gu, Seoul, 04763 Korea; 4 Division of Infectious Diseases, Department of Internal Medicine, Seoul National University Bundang Hospital, Seoul National University College of Medicine, 82 Gumi-ro, 173 beon-gil, Bundang-gu, Seongnam, 13620 Gyeonggi-do Korea; 5 Incheon Eun-Hye Hospital, Incheon, Korea; 6 Department of Economics, College of Political Science and Economics, Korea University, Seoul, Korea; 7 Division of Infectious Diseases, Department of Internal Medicine, Soonchunhyang University Seoul Hospital, Soonchunhyang University College of Medicine, Seoul, Korea; 8 Department of Internal Medicine, Inje University Sanggye-Paik Hospital, Seoul, Korea; 9 Division of Infectious Diseases, Department of Internal Medicine, Hallym University Sacred Heart Hospital, Anyang, Korea; 10 Division of Infectious Diseases, Department of Internal Medicine, College of Medicine, The Catholic University of Korea, Seoul, Korea

**Keywords:** Antibiotics, Stewardship, Resistance, Infectious diseases, Korea

## Abstract

**Background:**

Although non-teaching community hospitals form the majority of healthcare providers in South Korea, there is limited data on antibiotic usage in them. To evaluate the pattern of antibiotic usage and its appropriateness in hospitals with < 400 beds in South Korea.

**Methods:**

A multicentre retrospective study was conducted in 10 hospitals (six long-term care hospitals, three acute care hospitals, and one orthopaedic hospital), with < 400 beds in South Korea. We analysed patterns of antibiotic prescription in 2019, and their appropriateness in the participating hospitals. For the evaluation of the appropriateness of the prescription, 25 patients under antibiotic therapy were randomly selected at each hospital, over two separate periods. Due to the heterogeneity of their characteristics, the orthopaedics hospital was excluded from the analysis.

**Results:**

The most commonly prescribed antibiotics in long-term care hospitals was fluoroquinolone, followed by beta-lactam/beta-lactamase inhibitor (anti-pseudomonal). In acute care hospitals, these were third generation cephalosporin, followed by first generation cephalosporin, and second generation cephalosporin. The major antibiotics that were prescribed in the orthopedics hospital was first generation cephalosporin Only 2.3% of the antibiotics were administered inappropriately. In comparison, 15.3% of patients were prescribed an inappropriate dose. The proportion of inappropriate antibiotic prescriptions was 30.6% of the total antibiotic prescriptions.

**Conclusions:**

The antibiotic usage patterns vary between non-teaching community hospitals in South Korea. The proportion of inappropriate prescriptions exceeded 30% of the total antibiotic prescriptions.

**Supplementary Information:**

The online version contains supplementary material available at 10.1186/s13756-022-01082-2.

## Introduction

Antibiotics are one of the most significant discoveries in modern medical history. These drugs have saved innumerable lives; the timely administration of appropriate antibiotics, in particular, can reduce morbidity and mortality in patients with infectious diseases [1, 2]. Despite their obvious benefits, however, the misuse of antibiotics can lead to an increase in antibiotic-resistant pathogens as well as infectious diseases caused by these pathogens [3, 4]. Additionally, excessive use of antibiotics is related to *Clostridioides difficile* infections or adverse drug events, which may require additional hospital stay, consequently increasing healthcare costs [5, 6].

To reduce such harmful effects, optimize antibiotic use for infectious diseases, and minimize unnecessary use, the Antimicrobial Stewardship Program (ASP) was implemented globally [7]. The first step in implementing effective ASPs and assessing effective interventions is to understand the patterns of antibiotic use in hospitals [8, 9]. Therefore, the measurement of antibiotic prescriptions and the evaluation of antibiotic use form one of the core elements of ASP [7].

Most studies on antibiotic use and appropriateness of prescriptions in South Korea have been performed in large academic centres [10, 11]. Unfortunately, even though determining antibiotic use and prescription patterns is included in the Korean National Action Plan on antimicrobial resistance 2016–2020, there is limited data on the patterns of antibiotic use in non-teaching community hospitals [12]. Indeed, non-teaching community hospitals form the majority of healthcare providers in South Korea; in 2018, 94.5% of all hospitals had < 400 beds, with a mean bed size of 145 [13]. Therefore, we performed a study to evaluate the pattern of antibiotic usage and its appropriateness in non-teaching hospitals with < 400 beds in South Korea.

## Material and methods

### Study settings

To recruit hospitals, an e-mail containing information about the research was sent to non-teaching hospitals with < 400 beds via Infection Control Consulting Network in South Korea. Ten hospitals submitted applications for participation, and were included in our multicentre retrospective study. Of the participating hospitals, 6 (A-F) were long-term care hospitals, 3 (G-I) were acute care (general) hospitals, and 1 (J) was an orthopaedic hospital. The number of hospital beds ranged from 99 to 361, and 60% of the participating hospitals were in metropolitan areas (Seoul, Incheon, and Gyeonggi-do). The hospital names have been blinded to safeguard them from the possibility of unintended blame. The overall characteristics of the participating hospitals are listed in Table 1.

**Table 1:** Baseline characteristics for participated hospitals in the study

Hospital code	Classification	Location	Hospital beds	Number of human resources for ASP				Existence of laboratory for microbiological culture tests	Number of patients at the time of evaluation of appropriateness of antibiotic use			
				Physician	Nurse	Pharmacist	Staff in IT department		Hospitalization patients, March 22, 2021	Patients under antibiotic use, March 22, 2021	Hospitalizatio patients, May 31, 2021	Patients under antibiotic use, May 31, 2021
A	Long-term care hospital	Seoul	210	9	32	1	1	No	173	19	184	17
B	Long-term care hospital	Incheon	299	8	31	1	0	No	290	15	289	14
C	Long-term care hospital	Jeollabuk-do	99	2	6	1	1	No	63	1	62	0
D	Long-term care hospital	Seoul	229	7	45	1	3	No	213	12	214	6
E	Long-term care hospital	Daejeon	262	7	14	1	0	No	185	28	180	23
F	Long-term care hospital	Gyeonggi-do	256	8	53	1	2	No	111	3	128	10
G	Acute care hospital	Chungcheongnam-do	288	27	68	2	1	No	165	92	155	76
H	Acute care hospital	Incheon	195	17	99	1	1	No	122	54	108	55
I	Acute care hospital	Chungcheongbuk-do	361	70	194	4	3	Yes	312	169	309	175
J	Orthopedics hospital	Seoul	132	21	106	2	2	No	111	73	93	65

Abbreviations: ASP, antimicrobial stewardship program; IT, information technology

We analysed the antibiotic prescription patterns and their appropriateness in the participating hospitals. However, due to the heterogeneity of patient characteristics and antibiotic prescription patterns, hospital J (orthopaedics hospital) was excluded from the evaluation of the appropriateness of antibiotic prescription.

In this study, antibiotics were defined as medication with Anatomical Therapeutic Chemical class J01, which does not include antifungal, antituberculotic, antiparasitic, or antiviral agents. Systemic agents with oral or parenteral administration routes were included, whereas topical agents were excluded.

The study’s protocol was approved by the Institutional Review Board of Hanyang University, Seoul Hospital (IRB no. 2020-12-040). The requirement for an informed written consent from patients was waived because of the retrospective nature of the study.

### Analysis of antibiotic prescription patterns

Data on the monthly antibiotic prescriptions and patient-days for hospitalized patients were collected using electronic databases from each hospital. To avoid the effect of the coronavirus pandemic, data were collected from January to December 2019. The amount of antibiotic consumption was calculated using the defined daily dose (DDD) and days of therapy (DOT), and then standardized for 1000 patient-days [14]. We categorized antibiotic agents into 19 classes [10]: first generation cephalosporin (1G CEP), second generation cephalosporin (2G CEP), third generation cephalosporin (3G CEP), fourth generation cephalosporin, aminoglycoside, β-lactam/β-lactamase inhibitor (BL/BLI), BL/BLI (anti-pseudomonal), carbapenem, fluoroquinolone (FQ), glycopeptide, lincosamide, macrolide, metronidazole, oxazolidinone, penicillin, polymyxin, sulfonamide and trimethoprim, tetracycline, and tigecycline. Other antibiotics were excluded because they were rarely used.

### Evaluating the appropriateness of antibiotic prescription

To assess the appropriateness of antibiotic prescriptions, investigators in each hospital retrospectively collected information on patients with such prescriptions, according to the data collection forms. The collected data included patient information, information on antibiotic prescription, test results from microbiological cultures, and clinical information related to infectious diseases (Additional file 1: Supplement 1). The data were collected from two separate periods (22nd March 2021–21st April 2021 and 31st May 2021–30th June 2021) to minimise the effect of temporal bias. Investigators in each hospital collected cases that they treated in orders of the latest date, and then the assessment targets were randomly selected—a total of 25 patients in each hospital for each period; if the number of patients was less than 25, all patients were included. The assessment targets required that one patient be included only once; further, new-born babies (< 28 days old) were excluded.

The collected data were reviewed and the appropriateness of antibiotic prescriptions was evaluated by five specialists in infectious diseases (adult and paediatric). Data from two hospitals were assigned to each specialist. The appropriateness of antibiotic prescriptions was evaluated from three aspects: route of administration, dose, and class [15]. If the three aspects were ‘optimal’, the prescription was considered ‘optimal’; if only the route was ‘optimal’, and the dose and/or class was ‘suboptimal’ but not ‘inappropriate’, it was considered ‘suboptimal’; if even one aspect was ‘inappropriate’, it was classified as ‘inappropriate’.

Further, we assessed the appropriateness of the prescription by the patient: if all the prescribed antibiotics were evaluated as ‘optimal’ and there was no unnecessary combination, the antibiotic prescription for the patient was considered ‘optimal’; in case of at least one ‘suboptimal’ antibiotic prescription, but no ‘inappropriate’ antibiotic prescription, it was considered ‘suboptimal’. However, even if all antibiotic prescriptions were ‘optimal’ but the combination was unnecessary, it was considered ‘suboptimal’. If even one ‘inappropriate’ antibiotic prescription existed, it was considered as such (Table 2 and Additional file 2: Supplement 2).

**Table 2:** The standard for evaluation of the appropriateness of antibiotic use

	Optimal	Suboptimal	Inappropriate
Route of administration	A route that is recommended in the Sanford guide		A route that is not recommended in the Sanford guide
Dose	Within the range of recommendation of the Sanford guideline	the dose was significantly higher than the recommended amount	the dose was significantly lower than the recommended amount
Antibiotic class			
Treatment of infectious diseases	Antibiotics suggested in the guidelines, antibiotics that are susceptible to identified/possible pathogen	Antibiotic spectrum is too broad considering identified/possible pathogen^1^	Not included in optimal or suboptimal
Prophylaxis of surgical site infection	Adherent to the criteria of ‘The ninth nationwide evaluation of the appropriateness of surgical prophylactic antibiotics in Korean hospitals in 2020’		Not adherent to the criteria of ‘The ninth nationwide evaluation of the appropriateness of surgical prophylactic antibiotics in Korean hospitals in 2020’
Others	The designated infectious disease specialist judged it as ‘appropriate’		The designated infectious disease specialist judged it as ‘inappropriate’
Comprehensive evaluation for antibiotics	The route, dose, and class are ‘optimal’	The route is optimal AND dose and/or class is ‘suboptimal’ but not ‘inappropriate’	At least one ‘inappropriate’ exist for the route, dose, or class
Appropriateness of antibiotic prescription by the patient	All the prescribed antibiotics were evaluated as ‘optimal’ and there was no unnecessary combination	- At least one ‘suboptimal’ antibiotic prescription exists, but there was no ‘inappropriate’ antibiotic prescription - All the antibiotic prescriptions were ‘optimal’, but the combination was unnecessary	At least one ‘inappropriate’ antibiotic prescription exists

^1^Examples of antibiotic spectrum

< The rank of spectrum for beta-lactams >

- Rank 1: 3rd generation cephalosporins, Ureido/carboxy-penicillin

- Rank 2: Piperacillin/tazobactam, Ticarcillin/clavulanate, 4th generation cephalosporins, Anti-pseudomonal 3rd generation cephalosporins

- Rank 3: Ertapenem, Imipenem, Meropenem, Doripenem

< The rank of spectrum for anti-staphylococcal antibiotics >

- Rank 1: Nafcillin/Oxacillin, 1st generation cephalosporins,

- Rank 2: Vancomycin, Teicoplanin, Linezolid

We evaluated the appropriateness of the route of administration and dose using the Sanford guide to antimicrobial therapy [16]. Antibiotics administered via a route recommended in the Sanford guide were considered to have an ‘appropriate’ route of administration; the rest were considered ‘inappropriate’. The appropriateness of the dose was evaluated by analysing the patients’ renal function. Hence, the evaluation of antibiotics that needed a renal dose adjustment was conducted only when the results of the renal function test existed; cases without a result were considered ‘N/A’. If the dose was within the range of recommendation of the Sanford guide, it was considered ‘optimal’; if the dose was significantly higher than the recommended amount, it was considered ‘suboptimal’, and if the dose was significantly lower than the recommended amount, it was considered ‘inappropriate’.

The standards for the evaluation of the appropriateness of antibiotic classes differed according to the objectives of the prescription. If the antibiotic was prescribed for the treatment of an infectious disease, the appropriateness of the antibiotic class was assessed in accordance with antibiograms of isolated pathogens, clinical practice guidelines for infectious diseases, and the Sanford guide to antimicrobial therapy (Additional file 3: Supplement 3). Given that the antibiotic prescription might be evaluated as ‘inappropriate’ when the diagnosis of infectious diseases was incorrect, we also evaluated the appropriateness of the diagnosis; the standard for the evaluation of each infectious disease was distributed to infectious disease specialists (Additional file 3: Supplement 4). Prescribed antibiotics recommended in the guidelines and antibiotics susceptible to identified or possible pathogen were considered ‘optimal’; if the beta-lactam or anti-staphylococcal antibiotic spectrum was too broad considering identified or possible pathogen, it was evaluated as ‘suboptimal’; the rest were considered ‘inappropriate’. If the antibiotic was prescribed for prophylaxis in the prevention of surgical site infection, the appropriateness of antibiotic class was assessed using the criteria of ‘The ninth nationwide evaluation of the appropriateness of surgical prophylactic antibiotics in Korean hospitals in 2020’, led by the Health Insurance Review and Assessment Service [17]; if the prescribed antibiotics adhered to the criteria, they were considered ‘appropriate’; the rest were considered ‘inappropriate’. If the objective of the prescription did not belong to the treatment of infectious disease or prophylaxis for prevention of surgical site infection, the appropriateness was decided by the judgment of the designated infectious disease specialist (Table 2 and Additional file 2: Supplement 2).

### Statistical analysis

The results from evaluating the appropriateness of antibiotic prescriptions compared the differences between long-term care hospitals and acute care hospitals. All statistical analyses were conducted using SPSS version 24.0 for Windows (IBM Corp., Armonk, NY, USA). The categorical variables were analysed using the chi-square test or Fisher’s exact test, and the continuous variables were analysed using the Mann–Whitney U test. A two-tailed *P*-value of < 0.05 was considered statistically significant, for this study’s parameters.

## Results

### Antibiotic prescription patterns

The antibiotics being used in each hospital are described in Additional file 3: Supplement 5. The number of antibiotics used in each hospital ranged from 4 to 15 in long-term care hospitals, and from 24 to 36 in acute care hospitals. Twelve antibiotics were used in the orthopaedic hospital (hospital J).

The total amount of antibiotic consumption in 2019 ranged from 29.9 to 168.6 DDD/1000 patient-days (60.1-116.1 DOT/1000 patient-days) in long-term care hospitals, and from 546.7 to 674.9 DDD/1000 patient-days (577.5-616.6 DOT/1000 patient-days) in acute care hospitals. In the orthopaedic hospital, the total amount of antibiotic consumption was 410.1 DDD/1000 patient-days (563.9 DOT/1000 patient-days) (Additional file 3: Supplement 6).

**Fig. 1:**
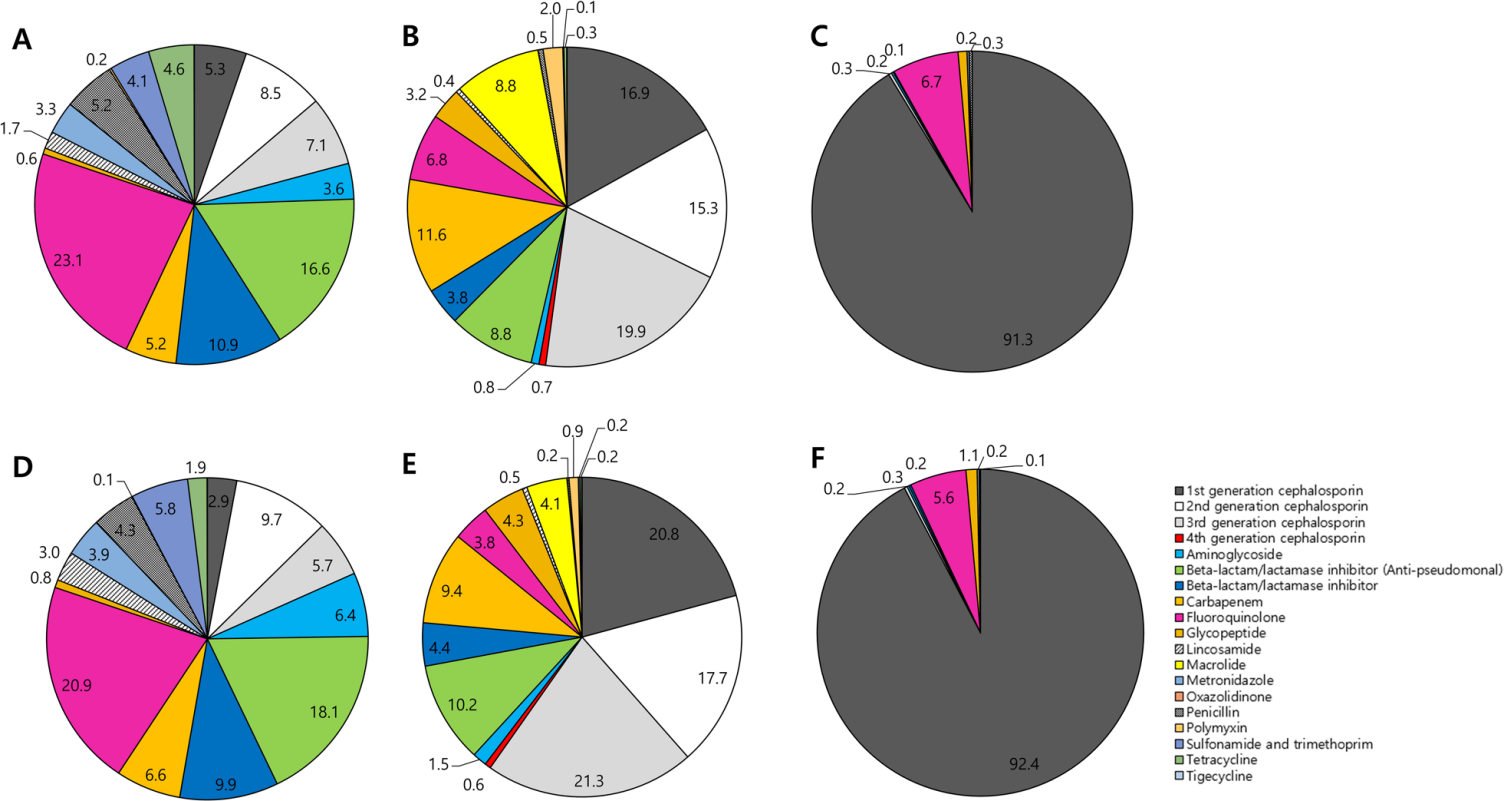
Differences in antibiotic usage pattern between long-term and acute care hospitals. **a** Long-term care hospitals, calculated by DDD/1000 patient-days. **b** Acute care hospitals, calculated by DDD/1000 patient-days. **c** An orthopaedic hospital, calculated by DDD/1000 patient-days. **d** Long-term care hospitals, calculated by DOT/1000 patient-days. **e** Acute care hospitals, calculated by DOT/1000 patient-days. **f** An orthopaedic hospital, calculated by DOT/1000 patient-days

**Fig. 2:**
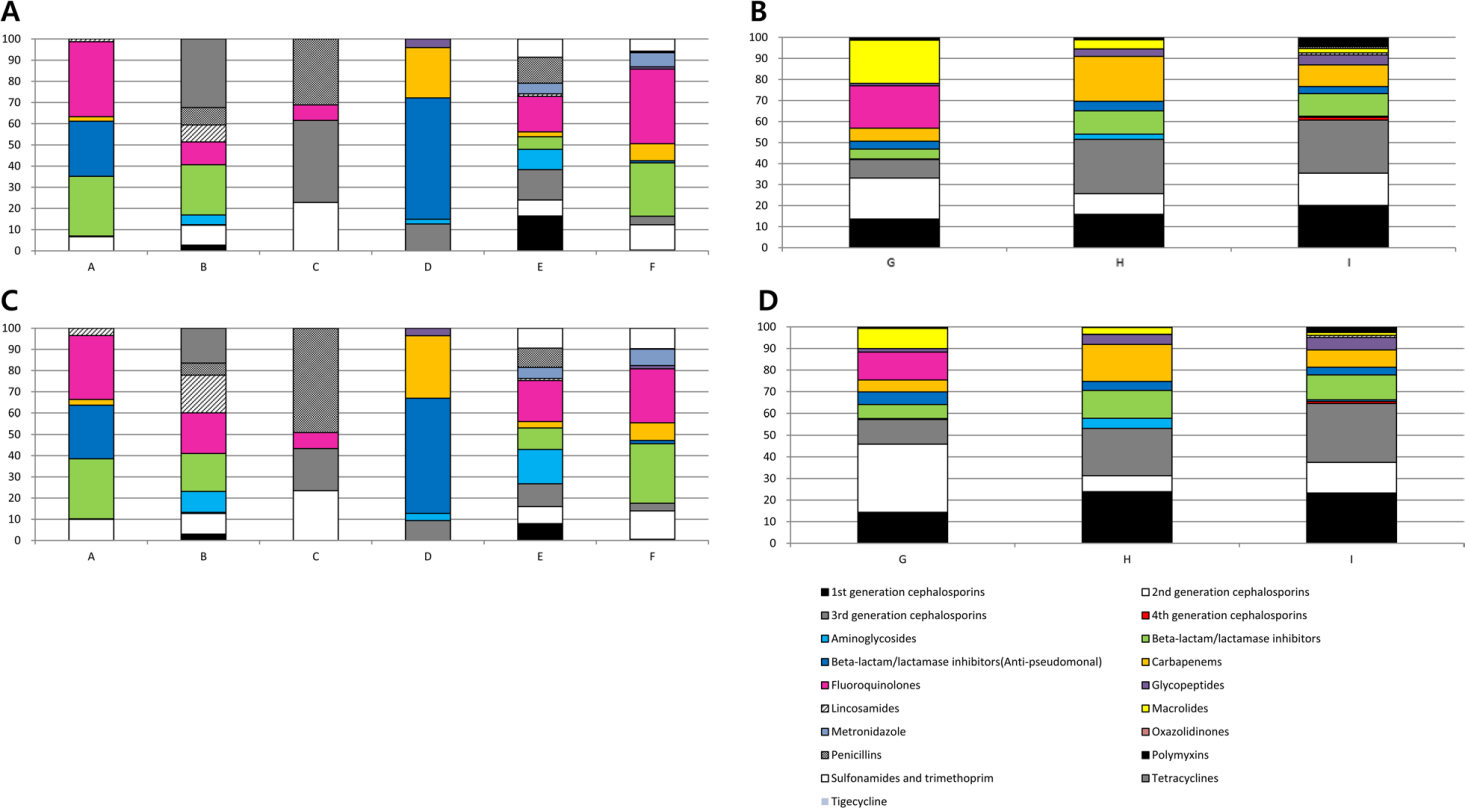
Difference in antibiotic usage pattern among hospitals. **a** Long-term care hospitals, calculated by DDD/1000 patient-days. **b** Acute care hospitals, calculated by DDD/1000 patient-days. **c** Long-term care hospitals, calculated by DOT/1000 patient-days. **d** Acute care hospitals, calculated by DOT/1000 patient-days

The most commonly prescribed antibiotic in long-term care hospitals was FQ (23.1% by DDD; 20.9% by DOT), followed by BL/BLI (anti-pseudomonal) (10.9% by DDD; 9.9% by DOT). In acute care hospitals, it was 3G CEP (19.9% by DDD; 21.3% by DOT), followed by 1G CEP (16.9% by DDD; 20.8% by DOT) and 2G CEP (15.3% by DDD; 17.7% by DOT). The proportion of carbapenem use in long-term and acute care hospitals was 5.2% by DDD (6.6% by DOT) and 11.6% by DDD (9.4% by DOT), respectively. The major antibiotics prescribed in the orthopaedic hospital was 1G CEP (91.3% by DDD; 92.4% by DOT) (Fig. 1). There were some differences in the frequency of antibiotics used among individual institutions, even within the same type of hospitals (Fig. 2).

### Appropriateness of antibiotic prescriptions

A total of 422 patients (272 from long-term and 150 from acute care hospitals) were recruited to study the appropriateness of antibiotic usage. A total of 569 antibiotics (384 from long-term and 185 from acute care hospitals) that were prescribed to these patients were evaluated.

Table 3 and Additional file 3: Supplement 7 show the baseline characteristics of the patients. The median age of patients from long-term care hospitals was 80.0 and higher than those from acute care hospitals (median 73.0) (*P* < 0.001). The proportion of females in long-term and acute care hospitals was 50.7% and 52.0%, respectively (*P* = 0.804). Data on renal function, which included the estimated glomerular filtration ratio, was available from the electronic medical record for 27.9% of the patients from long-term care hospitals, and 76.0% of the patients from acute care hospitals (*P* < 0.001). The proportion of patients with cognitive disorders (88.1% vs. 48.0%, *P* < 0.001) and in bedridden state (62.9% vs. 77.0%, *P* < 0.001) were higher in long-term care hospitals than in acute care hospitals. The proportion of patients who had results of microbiological culture with blood samples (12.1% vs. 68.5%, *P* < 0.001) and non-blood samples (13.6% vs. 69.6%, *P* < 0.001) were higher in acute care hospitals than in long-term care hospitals.

**Table 3:** Baseline characteristics for patients in the study for evaluation of appropriateness of antibiotic use

	Long-term care hospitals (N = 272)	Acute care hospitals (N = 150)	*P*-value	All hospitals (N = 422)
Age, median (IQR)	80.0 (72.0–86.0)	73.0 (61.0–82.0)	< 0.001	79.0 (67.0–85.0)
Female sex (%)	138 (50.7)	78 (52.0)	0.804	216 (51.2)
Ward type (%)				
General ward	260 (95.6)	118 (78.7)	< 0.001	378 (89.6)
Intensive care unit	12 (4.4)	32 (21.3)	-	44 (10.4)
Classification of department (%)				
Internal Medicine	68/270 (25.2)	97/149 (65.1)	< 0.001	165/419 (39.4)
Medical department (excluding internal medicine)	162/270 (60.0)	5/149 (3.4)	-	167/419 (39.9)
Surgical department	40/270 (14.8)	47/149 (31.5)	-	87/419 (20.8)
Data about renal function at EMR (%)				
Existence of result of CrCl	72 (26.5)	50 (33.3)	0.137	122 (28.9)
Existence of result of eGFR	76 (27.9)	114 (76.0)	< 0.001	190 (45.0)
Patients underwent renal replacement therapy (%)	24/270 (8.9)	6 (4.0)	0.062	30/420 (7.1)
Patients with cognitive disorder (%)	238/270 (88.1)	72 (48.0)	< 0.001	310/420 (73.8)
Ambulation status				
Ambulation, regardless of external support	17/270 (6.3)	69 (68.5)	< 0.001	86/420 (20.5)
Ambulation with wheelchair	45/270 (16.7)	25 (16.7)	-	70/420 (16.7)
Bed-ridden status	208/270 (77.0)	56 (37.3)	-	264/420 (62.9)
Microbiological culture test				
Existence of result of culture with blood sample	33 (12.1)	102/149 (68.5)	< 0.001	135/421 (32.1)
Existence of result of culture with non-blood sample	37 (13.6)	103/148 (69.6)	< 0.001	140/420 (33.3)

*IQR* interquartile range, *EMR* electronic medical record, *CrCl* creatinine clearance, *eGFR* estimated glomerular filtration rate

**Fig. 3:**
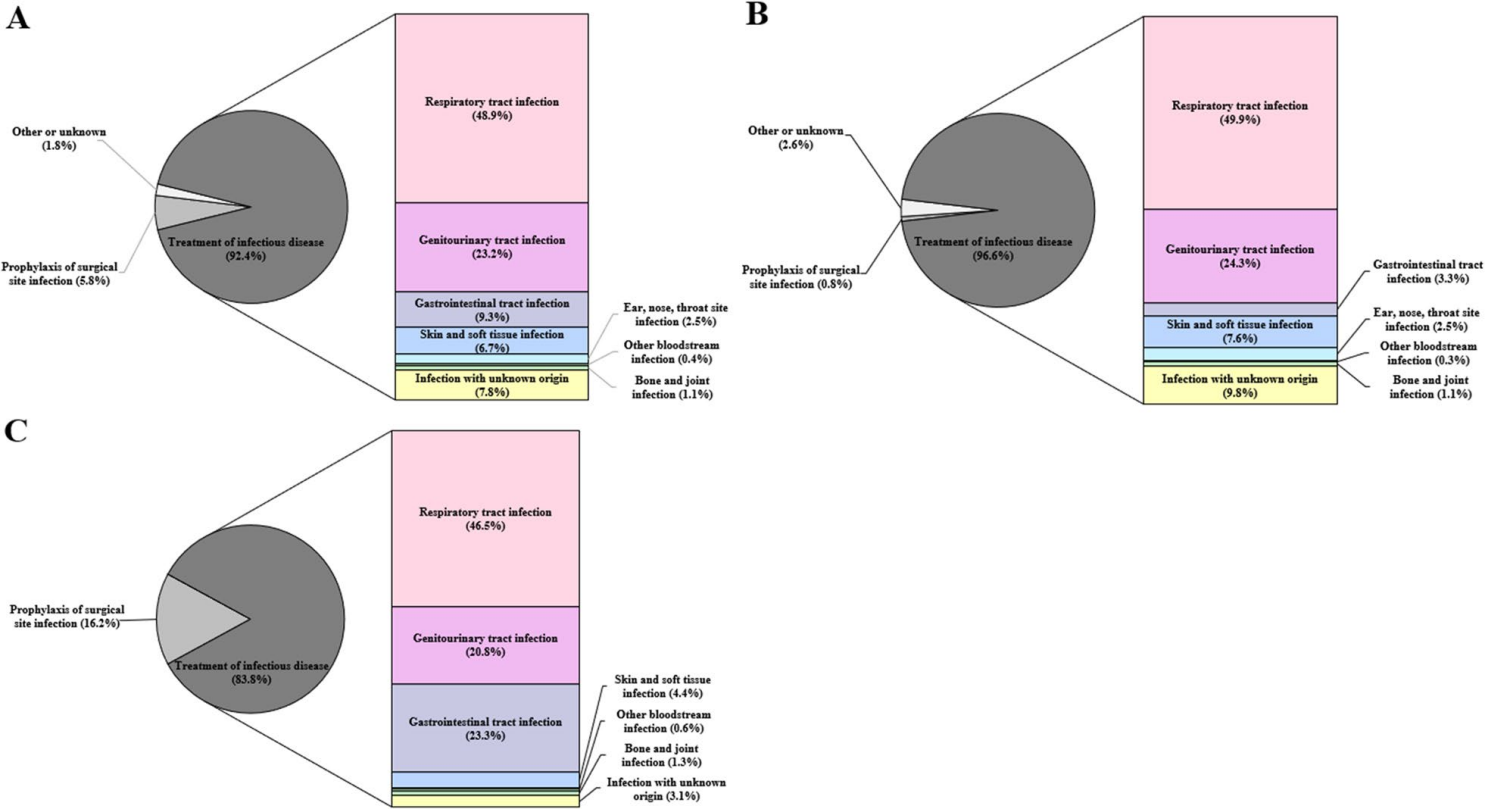
Purpose of antibiotic prescription. **a** All hospitals. **b** Long-term care hospitals. **c** Acute care hospitals

Figure 3, and Additional file 3: Supplements 8 and 9 show the characteristics of antibiotics evaluated in this study. The proportion of parenteral antibiotics were 63.8% in long-term care hospitals and 97.8% in acute care hospitals (*P* < 0.001). The majority of the evaluated antibiotics were prescribed to treat infectious diseases in both types of hospitals (96.3% vs. 83.8%, *P* < 0.001). The most common infectious disease was respiratory tract infections (48.5%), followed by genitourinary tract infections (23.0%), and gastrointestinal tract infections (9.2%).

Table 4 and Additional file 3: Supplement 10 show the appropriateness of the antibiotic prescriptions. Only 2.3% of antibiotics were administered inappropriately; the proportion of antibiotics with inappropriate routes of administration was not very different between long-term and acute care hospitals (2.6% vs. 1.6%, *P* = 0.562). In comparison, 15.3% patients were prescribed an inappropriate dose; the proportion of antibiotics with inappropriate doses were higher in long-term care hospitals than in acute care hospitals (16.1% vs. 13.5%, *P* < 0.001). As for the choice of antibiotics, 33.0% were evaluated as being inappropriate for treating infectious disease, and 45.5% were evaluated as inappropriate for the prophylaxis of surgical site infections. The proportion of inappropriate antibiotic choice for the treatment of infectious disease was higher in long-term care hospitals than in acute care hospitals (34.9% vs. 28.4%, *P* = 0.034). The proportion of inappropriate antibiotic prescriptions was 30.6% of the total antibiotic prescriptions, for the route, dose, and class of administration; 40.2% could not be evaluated due to insufficient data. In comparison, the proportion of patients with inappropriate antibiotic prescriptions was 32.0% of all patients who were prescribed antibiotics, and 38.2% could not be evaluated. The proportion of inappropriate antibiotic prescriptions (28.4% vs. 35.1%, *P* < 0.001) and that of patients with inappropriate antibiotics (31.3% vs. 33.3%, *P* < 0.001) were higher in acute care hospitals than in long-term care hospitals. The proportion of antibiotics that could not be evaluated (51.3% vs. 17.3%, *P* < 0.001) and those prescribed with antibiotics (48.2% vs. 20.0%, *P* < 0.001) were higher in long-term care hospitals.

**Table 4:** Appropriateness of antibiotic prescriptions

	Long-term care hospitals (N = 384)	Acute care hospitals (N = 185)	*P-*value	All hospitals (N = 569)
Route of administration (%)			0.562	
Appropriate	374 (97.4)	182 (98.4)		556 (97.7)
Inappropriate	10 (2.6)	3 (1.6)		13 (2.3)
Dose (%)			< 0.001	
Optimal	112 (29.2)	139 (75.1)		251 (44.1)
Suboptimal: excessively high dose	32 (8.3)	2 (1.1)		34 (6.0)
Inappropriate: excessively low dose	62 (16.1)	25 (13.5)		87 (15.3)
N/A	178 (46.4)	19 (10.3)		197 (34.6)
Antibiotic choice (%)				
Antibiotics for the treatment of infectious diseases			0.034	
Optimal	146/370 (39.5)^1^	82/155 (52.9)		228/525 (43.4)^1^
Suboptimal	60/370 (16.2)^1^	16/155 (10.3)		76/525 (14.5)^1^
Inappropriate	129/370 (34.9)^1^	44/155 (28.4)		173/525 (33.0)^1^
N/A	35/370 (9.5)^1^	13/155 (8.4)		48/525 (9.1)^1^
Antibiotics for the prophylaxis of surgical site infection			0.579	
Appropriate	1/3 (33.3)	17/30 (56.7)		18/33 (54.5)
Inappropriate	2/3 (66.7)	13/30 (43.3)		15/33 (45.5)
Antibiotics for other or unknown reasons			–	
Appropriate	0	0		0
Inappropriate	10/10 (100)	10/10 (100)		10/10 (100)
Appropriateness of antibiotic prescription, by each antibiotic (%)			< 0.001	
Optimal	49/384 (12.8)	76/185 (41.1)		125/569 (22.0)
Suboptimal	29/384 (7.6)	12/185 (6.5)		41/569 (7.2)
Inappropriate	109/384 (28.4)	65/185 (35.1)		174/569 (30.6)
N/A	197/384 (51.3)	32/185 (17.3)		229/569 (40.2)
Appropriateness of antibiotic prescription, by each patient (%)			< 0.001	
Optimal	29/272 (10.7)	57/150 (38.0)		86/422 (20.4)
Suboptimal: one or more antibiotics were suboptimal	26/272 (9.6)	9/150 (6.0)		35/422 (8.3)
Suboptimal: unnecessary combination therapy	1/272 (0.4)	4/150 (2.7)		5/422 (1.2)
Inappropriate	85/272 (31.3)	50/150 (33.3)		135/422 (32.0)
N/A	131/272 (48.2)	30/150 (20.0)		161/422 (38.2)

^1^One case was excluded because the data was insufficient

When cases with an inappropriate diagnosis were excluded, the proportion of inappropriate antibiotic prescriptions decreased to 21.2%, and that of patients with inappropriate antibiotic prescriptions decreased to 23.2% (Table 5). FQ (28.7%) and 3G CEP (19.5%) constituted the largest proportion of inappropriate antibiotic prescriptions (Additional file 3: Supplement 11). Of the common infectious diseases, the proportion of inappropriate antibiotic prescriptions was higher for respiratory tract infections (41.8%) than for genitourinary tract infections (39.2%) (Additional file 3: Supplement 12).

**Table 5:** Appropriateness of antibiotic prescriptions in infectious cases

	Long-term care hospitals	Acute care hospitals	*P-*value	All hospitals
Appropriateness of diagnosis for infectious diseases^1^ (%)				
By each antibiotic	282/370 (76.2)	119/155 (76.8)	0.078	401/525 (76.4)
By each patient	191/260 (73.5)	93/123 (75.6)	0.122	284/383 (74.2)
Appropriateness of antibiotic prescription, by each antibiotic (%)			< 0.001	
Optimal	49/370 (13.2)	65/155 (41.9)		114/525 (21.7)
Suboptimal	29/370 (7.8)	12/155 (7.7)		41/525 (7.8)
Inappropriate	98/370 (26.5)	54/155 (34.8)		152/525 (29.0)
N/A	194/370 (52.4)	24/155 (15.5)		218/525 (41.5)
Appropriateness of antibiotic, by each patient (%)			< 0.001	
Optimal	29/260 (11.2)	48/123 (39.0)		77/383 (20.1)
Suboptimal: one or more antibiotics were suboptimal	26/260 (10.0)	9/123 (7.3)		35/383 (9.1)
Suboptimal: unnecessary combination therapy	1/260 (0.4)	3/123 (2.4)		4/383 (1.0)
Inappropriate	75/260 (28.8)	41/123 (33.3)		116/383 (30.3)
N/A	129/260 (49.6)	22/123 (17.9)		151/383 (39.4)
Appropriateness of antibiotic prescription only in cases with the appropriate diagnosis, by each antibiotic (%)			< 0.001	
Optimal	49/282 (17.4)	64/119 (53.8)		113/401 (28.2)
Suboptimal	27/282 (9.6)	9/119 (7.6)		36/401 (9.0)
Inappropriate	53/282 (18.8)	32/119 (26.9)		85/401 (21.2)
N/A	153/282 (54.3)	14/119 (11.8)		167/401 (41.6)
Appropriateness of antibiotic prescription only in cases with the appropriate diagnosis, by each patient (%)			< 0.001	
Optimal	29/191 (15.2)	48/93 (51.6)		77/284 (27.1)
Suboptimal: one or more antibiotics were suboptimal	24/191 (12.6)	7/93 (7.5)		31/284 (10.9)
Suboptimal: unnecessary combination therapy	1/191 (0.5)	3/93 (3.2)		4/284 (1.4)
Inappropriate	43/191 (22.5)	23/93 (24.7)		66/284 (23.2)
N/A	94/191 (49.2)	12/93 (12.9)		106/284 (37.3)

^1^In the cases when antibiotics were prescribed for the treatment of infectious diseases

## Discussion

This study is the first to analyse the appropriateness of antibiotic usage patterns in non-teaching community hospitals in South Korea. Interestingly, antibiotic use was highly varied among individual hospitals. We found that hospital size was not a predictor of antibiotic use, consistent with previous studies [18, 19].

The antibiotic prescription pattern of acute care hospitals was similar to that of large academic hospitals in South Korea [10, 20]. The antibiotic prescription pattern of long-term care hospitals in South Korea has not been evaluated, and we found that the pattern was diverse varying across hospitals. Despite this, only three classes of antibiotics constituted more than half of the antibiotic agents prescribed in long-term as well as acute care hospitals. The most common drugs administered inappropriately were FQ and 3G CEP, which accounted for 48.2% of all inappropriately prescribed antibiotics (Additional file 3: Supplement 11). Considering the limitations of the workforce and infrastructure, implementing ASP focusing on drugs most frequently used, should be considered for non-teaching community hospitals.

The proportion of inappropriate antibiotic prescriptions exceeded 30% in this study, and was higher than the result (26.1%) of a previous study conducted in 75 hospitals—the majority of which were large academic hospitals—in South Korea [21]. Of the inappropriate antibiotic prescriptions for the treatment of infectious diseases, a substantial proportion seemed to be caused by inappropriate diagnosis. Indeed, the proportion of appropriate antibiotic prescriptions increased when cases with inappropriate diagnoses were excluded from the study. Previous studies have indicated that uncertainty of diagnosis was associated with an increase in antibiotic prescriptions, and that a large amount of inappropriate antibiotic prescriptions was attributable to deficits in diagnosis, rather than therapeutic knowledge [22, 23]. These results suggest that to improve antibiotic usage patterns, interventions focusing only on the judicious use of antibiotics are likely to be insufficient; efforts to enhance physicians’ diagnostic abilities will also be necessary.

Cases without sufficient data for the evaluation of the appropriateness were more often found in long-term care hospitals than acute care hospitals. Most of these cases did not have the results of creatinine levels, which is needed to adjust the antibiotic dose. This result might be associated with differences in the reimbursement system of national health insurance: diagnosis-related group reimbursements are mainly applied to long-term care hospitals, whereas fee-for-service reimbursement is primarily applied to acute care hospitals. Therefore, minimizing tests might have a positive effect for revenue in long-term care hospitals. This could also explain the phenomenon of not actively performing culture tests for identifying causative pathogens in long-term care hospitals. Therefore, to increase the appropriateness of antibiotic prescriptions, even in long-term care hospitals in South Korea, providing adequate reimbursement for essential tests to improve antibiotic use is necessary.

Our data demonstrated that the proportion of inappropriate antibiotic prescriptions was more common in prophylactic use for surgical site infection, than in therapeutic use for the treatment of infectious diseases. Prolonged antibiotic use after an operation was the major reason for ‘inappropriate’ antibiotic use (data not shown). In South Korea, the Health Insurance Review and Assessment Service, as part of the National Quality Assessment Program, has been assessing surgical prophylactic antibiotics in each hospital since 2007, to decrease the abuse and misuse of antibiotics [24]. As a result of this program, significant improvements have been reported in the administration of prophylactic antibiotics within one hour of surgical incision, and in the reduction in the use of inappropriate antibiotics, such as 3G CEP or aminoglycosides [24]. Since the inappropriate duration of antibiotic use was newly introduced in 2020, as a measurement of the program [17], the prolonged use of surgical prophylactic antibiotics might reduce in the near future.

Additionally, there was not enough workforce to ensure the successful implementation of ASP in the participant hospitals: there was a limited number of infectious disease specialists (IDS) (only one hospital employed IDS, data not shown), as well as pharmacists and staff for information technology (Table 1). Given that the amount of antibiotic use in non-teaching community hospitals is similar to those in large hospitals, investment to increase the staffing for such a workforce is necessary [18, 19]. IDS play a major role in managing patients with various infectious diseases, and in the organization of ASP in each hospital [25, 26]. However, despite the importance of their role, the number of IDS in South Korea remains insufficient; therefore, it is difficult to find them in non-teaching community hospitals. In 2020, there were only 242 IDS, most of whom were employed in large hospitals [27, 28]. Given the shortage of workforce, less labour-intensive ASP strategies, such as syndrome-based intervention against common infectious diseases, may be a potential solution to improve the appropriateness of antibiotic use in non-teaching community hospitals [29]. Furthermore, a collaborative, consultation network focused on ASP implementation in non-teaching community hospitals, and supported by large hospitals with sufficient infrastructure for ASP, is necessary in South Korea [30]. Fortunately, the recently announced Korean National Action Plan on Antimicrobial Resistance 2021–2025 includes a plan to conduct a pilot project about the consultation network of ASP as a countermeasure for antimicrobial resistance in non-teaching community hospitals [31].

There are several potential limitations in the present study. First, we did not collect data on patients’ clinical information. Therefore, the appropriateness of antibiotic use could not be determined in some cases. Second, a large proportion of antibiotics and patients could not be fully evaluated because of insufficient data. To overcome this shortcoming, the appropriateness of antibiotics was presented in the subdivision as well: route of administration, dose, and antibiotic choice. Third, the generalizability of our results is limited because the characteristics of the nine hospitals may not be representative of all non-teaching community hospitals in South Korea. Indeed, there are more than 3000 non-teaching community hospitals in South Korea. Additionally, selection bias might exist because we recruited all hospitals that voluntarily submitted applications for participation. Considering the healthcare system in South Korea, which is well controlled by National Health Insurance, and considering the limited workforce for ASP in primary care or long-term care hospitals, the situation might not be different in other non-teaching community hospitals [27]. Finally, the study did not properly evaluate the proportion of patients receiving antibiotics from all inpatients during the study period, which would better reflect the burden of antibiotic use.

## Conclusion

The antibiotic usage patterns vary among non-teaching community hospitals in South Korea. The proportion of inappropriate antibiotic prescriptions exceeds 30% of the total antibiotic prescriptions, and the inaccurate diagnosis seems to be positively associated with inappropriate antibiotic prescriptions. Given the limited workforce and infrastructure, customized strategies for these hospitals and national-level support are necessary to improve ASP in non-teaching community hospitals in South Korea.

## Supplementary Information

**Additional file 1: Supplement 1**. Data collection form (for investigators in each hospital).

**Additional file 2: Supplement 2**. Evaluation of appropriateness of antibiotic prescription (for infectious diseases specialists).

**Additional file 3: Supplement 3–12**.

## Data Availability

The datasets used and/or analyzed during the current study are available from the corresponding author on reasonable request.

## Abbreviations

ASP: Antimicrobial stewardship program
DDD: Defined daily dose
DOT: Days of therapy
1G CEP: First generation cephalosporin
2G CEP: Second generation cephalosporin
3G CEP: Third generation cephalosporin
BL/BLI: β-Lactam/β-lactamase inhibitor
FQ: Fluoroquinolone
IDS: Infectious disease specialists
