# Supplementary material for: Prescriptions patterns and appropriateness of usage of antibiotics in non-teaching community hospitals in South Korea: a multicentre retrospective study
Source: Antimicrob Resist Infect Control. 2022 Feb 22;11:40. doi: 10.1186/s13756-022-01082-2 (PMC8861602; doi:10.1186/s13756-022-01082-2)
Supplement: Supplementary file 1 — Additional file 1: Supplement 1. Data collection form (for investigators in each hospital). [file 13756_2022_1082_MOESM1_ESM.docx]

**Data Collection Form (for investigators in each hospital)**

| **Patient information** | |
| --- | --- |
| **Hospital name/Patient number** : | |
| **Date of evaluation** :□□□□/□□/□□ (YY-MM-DD) | |
| **Patient name** : (Initial)___________ | |
| **Date of birth** : □□□□/□□ (YY-MM) | Sex : ○Male ○Female |
| **Date of admission** :□□□□/□□/□□ (YY-MM-DD) | |
| **Type of ward** | ○ General ward ○ Intensive care unit |
| **Department of admission**  ○ Internal Medicine ○ Pediatrics ○ General surgery ○ Plastic surgery ○ Neurosurgery  ○ Orthopedics ○ Chest surgery ○ Family medicine ○ Urology ○ Obstetrics & gynecology  ○ Neurology ○ Opthalmology ○ Emergency medicine ○ Otolaryngology  ○ Rehabilitation ○ Psychiatry ○ Dermatology ○ Others (___________________________) | |
| **Body weight**^1)^: ___________kg (○ Not applicable) | |
| **CrCl**^1)^ : ___________mL/min (○ Not applicable) | **eGFR**^1)^ : __________mL/min (○ Not applicalbe) |
| **Underwent renal replacement therapy** | ○ Yes ○ No |
| **Existence of cognitive disability** | ○ Yes ○ No |
| **Ambulatory status** | ○ Ambulation ○ Wheelchair ambulation  ○ Bed-ridden state |

1. *In the case of weight, CrCl, and eGFR, the results of the nearest date from the time of evaluation are recorded among the results within 3 months.*

| **Antibiotic prescription information** | | | |
| --- | --- | --- | --- |
| **Antibiotics - 1** | | | |
| **Antibiotics (component name)**^1)^ | |  | |
| **Route of administration** | | ○ Parenteral ○ Per oral ○ Others (_______________) | |
| **Dose** | | Dose per administration : __________________(mg)  Administration number/day^2)^ : ○0.33 ○0.5 ○1 ○2 ○ 3  ○4 ○5 ○6 ○Others (_________________) | |
| **Objectives of antibiotic prescription**  **(Choose one of the three)** | **Treatment of infectious diseases** | | ○ Genitourinary tract infection ○ Skin & soft tissue infection  ○ Respiratory tract infection ○ Gastrointestinal tract infection  ○ Ear, nose, throat infection ○ Other bloodstream infection^3)^ ○ Bone and joint infection ○ Infection with unknown origin  ○ Others (___________________) |
|  |  |  | ○ Diagnosed at other hospital  ○ Diagnosed at the present hospital |
|  | **Prophylaxis for prevention of surgical site infection** | | ○ Date of operation: □□□□/□□/□□ (YY-MM-DD)  ○ Type of operation  □ Stomach □ Colorectal □ Gallbldder  □ Total hip replacement □ Total knee replacement  □ Hystetectomy □ Cesarean section  □ Open heart surgery □ Brain surgery  □ Prostatectomy □ Cataract □ Thyroid  □ Breast □ Spine □ Shoulder  □ Throat □ Hernia □ Glaucoma  □ Lung □ Fracture □ Blood vessel  □ Appendectomy  □ Implantable cardioverter defibrillator insertion  □ Others (________________) |
|  | **Others** | | ○ Others (___________________)  ○ The purpose of prescription is unknown |
| **Antibiotics - 2** | | | |
| **Antibiotics (component name)**^1)^ | |  | |
| **Route of administration** | | ○ Parenteral ○ Per oral ○ Others (_______________) | |
| **Dose** | | Dose per administration : __________________(mg)  Administration number/day^2)^ : ○0.33 ○0.5 ○1 ○2 ○ 3  ○4 ○5 ○6 ○Others (_________________) | |
| **Objectives of antibiotic prescription**  **(Choose one of the three)** | **Treatment of infectious diseases** | | ○ Genitourinary tract infection ○ Skin & soft tissue infection  ○ Respiratory tract infection ○ Gastrointestinal tract infection  ○ Ear, nose, throat infection ○ Other bloodstream infection^3)^ ○ Bone and joint infection ○ Infection with unknown origin  ○ Others (___________________) |
|  |  |  | ○ Diagnosed at other hospital  ○ Diagnosed at the present hospital |
|  | **Prophylaxis for prevention of surgical site infection** | | ○ Date of operation: □□□□/□□/□□ (YY-MM-DD)  ○ Type of operation  □ Stomach □ Colorectal □ Gallbldder  □ Total hip replacement □ Total knee replacement  □ Hystetectomy □ Cesarean section  □ Open heart surgery □ Brain surgery  □ Prostatectomy □ Cataract □ Thyroid  □ Breast □ Spine □ Shoulder  □ Throat □ Hernia □ Glaucoma  □ Lung □ Fracture □ Blood vessel  □ Appendectomy  □ Implantable cardioverter defibrillator insertion  □ Others (________________) |
|  | **Others** | | ○ Others (___________________)  ○ The purpose of prescription is unknown |
| **Antibiotics - 3** | | | |
| **Antibiotics (component name)**^1)^ | |  | |
| **Route of administration** | | ○ Parenteral ○ Per oral ○ Others (_______________) | |
| **Dose** | | Dose per administration : __________________(mg)  Administration number/day^2)^ : ○0.33 ○0.5 ○1 ○2 ○ 3  ○4 ○5 ○6 ○Others (_________________) | |
| **Objectives of antibiotic prescription**  **(Choose one of the three)** | **Treatment of infectious diseases** | | ○ Genitourinary tract infection ○ Skin & soft tissue infection  ○ Respiratory tract infection ○ Gastrointestinal tract infection  ○ Ear, nose, throat infection ○ Other bloodstream infection^3)^ ○ Bone and joint infection ○ Infection with unknown origin  ○ Others (___________________) |
|  |  |  | ○ Diagnosed at other hospital  ○ Diagnosed at the present hospital |
|  | **Prophylaxis for prevention of surgical site infection** | | ○ Date of operation: □□□□/□□/□□ (YY-MM-DD)  ○ Type of operation  □ Stomach □ Colorectal □ Gallbldder  □ Total hip replacement □ Total knee replacement  □ Hystetectomy □ Cesarean section  □ Open heart surgery □ Brain surgery  □ Prostatectomy □ Cataract □ Thyroid  □ Breast □ Spine □ Shoulder  □ Throat □ Hernia □ Glaucoma  □ Lung □ Fracture □ Blood vessel  □ Appendectomy  □ Implantable cardioverter defibrillator insertion  □ Others (________________) |
|  | **Others** | | ○ Others (___________________)  ○ The purpose of prescription is unknown |

1. *The antibiotic is recorded by the component name (e.g. ciprofloxacin O, fluoroquinolone X, cirok X).*
2. *If administered once every 3 days, the number of administrations is 0.33 times, and if administered once every 2 days, the number of administrations is 0.5 times.*
3. *An infection in which identified bacteria exist in blood culture, but the cause is unknown.*

| **Microbiological culture test results (Please fill it out only if there is a result)^1)^** | | |
| --- | --- | --- |
| **Conduction of blood culture** | | ○ Yes ○ No |
| **Conduction of culture with other specimens (sputum, urine, pus, cerebrospinal fluid, pleural fluid, ascites, etc)** | | ○ Yes ○ No |
| **Microorganism - 1 (Please fill it out only if there is a result)^1)^** | | |
| **Specimen** | □ Blood □ Sputum □ Urine □ Pus □ Cerebrospinal fluid  □ Pleural fluid □ Ascites □ Others (______________________) | |
| **Isolated microorganism** | □ *Staphylococcus aureus*  □ *Entorococcus* spp.  □ Coagulase-negative Staphylococci  □ Enterobacteriales (*Escherichia coli, Klebsiella pneumoniae, Proteus spp., Serratia spp., Citrobacter spp*.)  □ *Pseudomonas aeruginosa*  □ *Acinetobacter baumanii*  □ Not applicable (No results for the bacteria mentioned above) | |
| **Existence of resistance** | Oxacillin resistance ○ Yes ○ No ○ Not applicable  Vancomycin resistance ○ Yes ○ No ○ Not applicable  ESBL production ○ Yes ○ No ○ Not applicable  Carbapenem^2)^ resistance ○ Yes ○ No ○ Not applicable | |
| **Microorganism - 2 (Please fill it out only if there is a result)^1)^** | | |
| **Specimen** | □ Blood □ Sputum □ Urine □ Pus □ Cerebrospinal fluid  □ Pleural fluid □ Ascites □ Others (______________________) | |
| **Isolated microorganism** | □ *Staphylococcus aureus*  □ *Entorococcus* spp.  □ Coagulase-negative Staphylococci  □ Enterobacteriales (*Escherichia coli, Klebsiella pneumoniae, Proteus spp., Serratia spp., Citrobacter spp*.)  □ *Pseudomonas aeruginosa*  □ *Acinetobacter baumanii*  □ Not applicable (No results for the bacteria mentioned above) | |
| **Existence of resistance** | Oxacillin resistance ○ Yes ○ No ○ Not applicable  Vancomycin resistance ○ Yes ○ No ○ Not applicable  ESBL production ○ Yes ○ No ○ Not applicable  Carbapenem^2)^ resistance ○ Yes ○ No ○ Not applicable | |
| **Microorganism - 3 (Please fill it out only if there is a result)^1)^** | | |
| **Specimen** | □ Blood □ Sputum □ Urine □ Pus □ Cerebrospinal fluid  □ Pleural fluid □ Ascites □ Others (______________________) | |
| **Isolated microorganism** | □ *Staphylococcus aureus*  □ *Entorococcus* spp.  □ Coagulase-negative Staphylococci  □ Enterobacteriales (*Escherichia coli, Klebsiella pneumoniae, Proteus spp., Serratia spp., Citrobacter spp*.)  □ *Pseudomonas aeruginosa*  □ *Acinetobacter baumanii*  □ Not applicable (No results for the bacteria mentioned above) | |
| **Existence of resistance** | Oxacillin resistance ○ Yes ○ No ○ Not applicable  Vancomycin resistance ○ Yes ○ No ○ Not applicable  ESBL production ○ Yes ○ No ○ Not applicable  Carbapenem^2)^ resistance ○ Yes ○ No ○ Not applicable | |

1. *Screening test results for infection control (stool VRE, etc.) are not included.*
2. *It includes Imipenem, Meropenem, Doripenem, and Ertapenem*

| **Evaluation of infectious diseases** | |
| --- | --- |
| **Existence of indwelling device** | □ Urinary catheter □ Central venous catheter^1)^  □ Ventilator □ Nasogastric tube □ Not applicable |
| **Fever (≥37.8℃)** | ○ Not existence ○ Within 3 days  ○ Before 4-7 days ○ Before 7-14 days |
| **Leukocytosis (WBC ≥14,000 OR bands ≥6%)** | ○ Not existence ○ Within 3 days  ○ Before 4-7 days ○ Before 7-14 days |
| **Hyoptension (sBP < 90 mmHg OR reduced >30 mmHg from baseline)** | ○ Not existence ○ Within 3 days  ○ Before 4-7 days ○ Before 7-14 days |
| **Acute change of mental status** | ○ Yes ○ No |
| **Sign/symptoms relevant to genitourinary tract infection** | ○ Existence ○ Absence |
|  | □ Newly developed or aggravated dysuria  □ Newly developed or aggravated frequency  □ Newly developed or aggravated urgency  □ Newly developed or aggravated incontinence  □ Gross hematuria □ Gross pyuria  □ Suprapubic tenderness □ Costovertebral tenderness  □ Acute pain/swelling or tenderness of the testes and/or penis  □ Bacteriuria (detected at urinalysis or culture) |
|  | Others (_________________________________) |
| **Sign/symptoms relevant to skin & soft tissue infection** | ○ Existence ○ Absence |
|  | Site (_________________________________)  □ Heating sense □ Pain/tenderness  □ Redness □ Serous or purulent discharge  □ Swelling |
|  | Others (_________________________________) |
| **Sign/symptoms relevant to bone & joint infection** | ○ Existence ○ Absence |
|  | Site (_________________________________)  □ Heating sense □ Pain/tenderness  □ Redness □ Serous or purulent discharge  □ Swelling |
|  | Others (_________________________________) |
| **Sign/symptoms relevant to respiratory tract infection** | ○ Existence ○ Absence |
|  | □ Newly developed or aggravated cough  □ Newly developed or aggravated sputum  □ Oxygen saturation <94% (room air) or reduced >3% from baseline저하  □ Pleuritic chest pain □ Respiratory rate ≥25/min  □ Newly developed or aggravated crackle on lung examination  □ Findings with suspected pneumonia at chest radiography |
|  | Others (_________________________________) |
| **Sign/symptoms relevant to gastrointestinal tract infection** | ○ Existence ○ Absence |
|  | □ Diarrhea (three or more unformed stools in 24h period)  □ Vomiting (two or more in 24h period)  □ Abdominal pain  □ Causative microorganism was isolated from stool specimen□ Positive result from C. difficile associated tests (CD culture, CD toxin ELISA/PCR, etc)  □ Pseudomembrane was found at endoscopy |
|  | Others (_________________________________) |
| **Sign/symptoms relevant to ear, nose, throat infection** | ○ Existence ○ Absence |
|  | □ Diagnosed ear infection through examination by a physician □ Newly developed or aggravated discharge from one or both ears  □ Diagnosed sinusitis through examination by a physician  □ Existence of swelling or discharge on tonsil  □ Tenderness on cervical lymph nodes |
|  | Others (_________________________________) |

1. *It includes PICC, Double lumen catheter, Perm catheter, and Hickman catheter*
