# Supplementary material for: Prescriptions patterns and appropriateness of usage of antibiotics in non-teaching community hospitals in South Korea: a multicentre retrospective study
Source: Antimicrob Resist Infect Control. 2022 Feb 22;11:40. doi: 10.1186/s13756-022-01082-2 (PMC8861602; doi:10.1186/s13756-022-01082-2)
Supplement: Supplementary file 2 — Additional file 2: Supplement 2. Evaluation of appropriateness of antibiotic prescription (for infectious diseases specialists). [file 13756_2022_1082_MOESM2_ESM.docx]

**Evaluation of appropriateness of antibiotic prescription**

**(for infectious diseases specialists)**

| **Hospital name/Patient number** |  | |
| --- | --- | --- |
| **Antibiotics - 1** | | |
| **Route of administration** | ○ Appropriate  ○ Inappropriate (a route that is not recommended e.g. irrigation) | |
| **Dose** | ○ Optimal ○ Suboptimal: overdose ○ Inappropriate: underdose  ○ Not accessible: absence of information about renal function | |
| **Class** | **Treatment of infectious diseases** | **Appropriateness of diagnosis of infectious diseases** |
|  |  | ○ Appropriate ○ Inappropriate |
|  |  | ○ Optimal: antibiotics suggested in the guidelines, antibiotics that are susceptible to identified/possible pathogen  ○ Suboptimal: antibiotic spectrum is too broad considering identified/possible pathogen^1)^  ○ Inappropriate (___________________)  ○ Not accessible (___________________) |
|  | **Prophylaxis of surgical site infection** | ○ Appropriate  ○ Inappropriate (___________________)  ○ Not accessible (___________________) |
|  | **Others** | The objectives of antibiotic prescription (___________________)  ○ Appropriate  ○ Inappropriate (___________________) |
| **Comprehensive evaluation for antibotics -1** | ○ Optimal: the route, dose, and class are ‘optimal’  ○ Suboptimal: the route is optimal AND dose and/or class is ‘suboptimal’ but not ‘inappropriate’  ○ Inappropriate: at least one ‘inappropriate’ exist for the route, dose, or class  ○ Not accessible | |
| **Antibiotics - 2** | | |
| **Route of administration** | ○ Appropriate  ○ Inappropriate (a route that is not recommended e.g. irrigation) | |
| **Dose** | ○ Optimal ○ Suboptimal: overdose ○ Inappropriate: underdose  ○ Not accessible: absence of information about renal function | |
| **Class** | **Treatment of infectious diseases** | **Appropriateness of diagnosis of infectious diseases** |
|  |  | ○ Appropriate ○ Inappropriate |
|  |  | ○ Optimal: antibiotics suggested in the guidelines, antibiotics that are susceptible to identified/possible pathogen  ○ Suboptimal: antibiotic spectrum is too broad considering identified/possible pathogen^1)^  ○ Inappropriate (___________________)  ○ Not accessible (___________________) |
|  | **Prophylaxis of surgical site infection** | ○ Appropriate  ○ Inappropriate (___________________)  ○ Not accessible (___________________) |
|  | **Others** | The objectives of antibiotic prescription (___________________)  ○ Appropriate  ○ Inappropriate (___________________) |
| **Comprehensive evaluation for antibotics -1** | ○ Optimal: the route, dose, and class are ‘optimal’  ○ Suboptimal: the route is optimal AND dose and/or class is ‘suboptimal’ but not ‘inappropriate’  ○ Inappropriate: at least one ‘inappropriate’ exist for the route, dose, or class  ○ Not accessible | |
| **Antibiotics - 3** | | |
| **Route of administration** | ○ Appropriate  ○ Inappropriate (a route that is not recommended e.g. irrigation) | |
| **Dose** | ○ Optimal ○ Suboptimal: overdose ○ Inappropriate: underdose  ○ Not accessible: absence of information about renal function | |
| **Class** | **Treatment of infectious diseases** | **Appropriateness of diagnosis of infectious diseases** |
|  |  | ○ Appropriate ○ Inappropriate |
|  |  | ○ Optimal: antibiotics suggested in the guidelines, antibiotics that are susceptible to identified/possible pathogen  ○ Suboptimal: antibiotic spectrum is too broad considering identified/possible pathogen^1)^  ○ Inappropriate (___________________)  ○ Not accessible (___________________) |
|  | **Prophylaxis of surgical site infection** | ○ Appropriate  ○ Inappropriate (___________________)  ○ Not accessible (___________________) |
|  | **Others** | The objectives of antibiotic prescription (___________________)  ○ Appropriate  ○ Inappropriate (___________________) |
| **Comprehensive evaluation for antibotics -1** | ○ Optimal: the route, dose, and class are ‘optimal’  ○ Suboptimal: the route is optimal AND dose and/or class is ‘suboptimal’ but not ‘inappropriate’  ○ Inappropriate: at least one ‘inappropriate’ exist for the route, dose, or class  ○ Not accessible | |
| **Appropriateness of antibiotic prescription by the patient** | | |
| ○ Optimal: all the prescribed antibiotics were evaluated as ‘optimal’ and there was no unnecessary combination  ○ Suboptimal  - at least one ‘suboptimal’ antibiotic prescription exists, but there was no ‘inappropriate’ antibiotic prescription  - all the antibiotic prescriptions were ‘optimal’, but the combination was unnecessary.  ○ Inappropriate: at least one ‘inappropriate’ antibiotic prescription exist  ○ Not accessible | | |

*^1)^ Examples of antibiotic spectrum*

***<The rank of spectrum for beta-lactams*** *(Ref: Clin Microbiol Infect 2015; 21: 649)>*

*- Rank 1: 3^rd^ G cephalosporins, Ureido/carboxy-penicillin*

*- Rank 2: Piperacillin/tazobactam, Ticarcillin/clavulanate, 4^th^ G cephalosporins, Anti-pseudomonal 3^rd^ G cephalosporins*

*- Rank 3: Ertapenem, Imipenem, Meropenem, Doripenem*

***<The rank of spectrum for anti-staphylococcal antibiotics****. (Ref: Johns Hopkins ABx guide)>*

*- Rank 1: Nafcillin/Oxacillin, 1^st^ G cephalosporins,*

*- Rank 2: Vancomycin, Teicoplanin, Linezolid*
