# Supplementary material for: Prescriptions patterns and appropriateness of usage of antibiotics in non-teaching community hospitals in South Korea: a multicentre retrospective study
Source: Antimicrob Resist Infect Control. 2022 Feb 22;11:40. doi: 10.1186/s13756-022-01082-2 (PMC8861602; doi:10.1186/s13756-022-01082-2)
Supplement: Supplementary file 3 — Additional file 3: Supplement 3–12. [file 13756_2022_1082_MOESM3_ESM.docx]

Supplement 3. Guidelines that were used as reference for the evaluation appropriateness of antibiotic prescriptions

| Infection site | Reference guidelines |
| --- | --- |
| Genitourinary tract infection | 1. Kang C-I, et al. Clinical Practice Guidelines for the Antibiotic Treatment of Community-Acquired Urinary Tract Infections. Infect Chemother 2018; 50: 67-100 |
| Skin and soft tissue infection | 1. Kwak YG, et al. Clinical Guidelines for the Antibiotic Treatment for Community-Acquired Skin and Soft Tissue Infection. Infect Chemother 2017; 49: 301-325 2. Stevens DL, et al. Practice guidelines for the diagnosis and management of skin and soft tissue infections: 2014 update by the Infectious Diseases Society of America. Clin Infect Dis 2014; 59: e10-52 |
| Bone and joint infection | 1. Clinical Guidelines for the Antimicrobial Treatment of Bone and Joint Infections in Korea. Infect Chemother 2014; 46: 125-138 |
| Respiratory tract infection | 1. Yoon YK, et al. Guidelines for the Antibiotic Use in Adults with Acute Upper Respiratory Tract Infections. Infect Chemother 2017; 49: 326-352 2. Lee MS, et al. Guideline for Antibiotic Use in Adults with Community-acquired Pneumonia. Infect Chemother 2018; 50: 160-198 3. Korea Disease Control and Prevention Agency. Guideline for acute upper respiratory infection among children 2017 |
| Gastrointestinal tract infection | 1. Kim YJ, et al. Guideline for the Antibiotic Use in Acute Gastroenteritis. Infect Chemother 2019; 51: 217-243 2. Shane AL, et al. 2017 Infectious Diseases Society of America Clinical Practice Guidelines for the Diagnosis and Management of Infectious Diarrhea. Clin Infect Dis 2017; 65: 1963-1973 3. Solomkin JS, et al. Diagnosis and management of complicated intra-abdominal infection in adults and children: guidelines by the Surgical Infection Society and the Infectious Diseases Society of America. Clin Infect Dis 2010; 50: 133-164 4. Mazuski JE, et al. The Surgical Infection Society Revised Guidelines on the Management of Intra-Abdominal Infection. Surg Infect (Larchmt) 2017; 18: 1-76 5. Debast SB, et al. European Society of Clinical Microbiology and Infectious Diseases: update of the treatment guidance document for Clostridium difficile infection. Clin Microbiol Infect 2014;20 Suppl 2: 1-26 |
| Ear, nose, throat site infection | 1. Lee H-J, et al. The diagnosis and management of otitis media in children. J Korean Med Assoc 2015; 58: 635-644 |

Supplement 4. The standard for evaluation of each infectious disease

| **Genitourinary tract infection** | |
| --- | --- |
| Absence of indwelling urinary catheter (Satisfaction with one or more of 1-3) | 1. Recently aggravated dysuria OR acute pain/swelling or tenderness of the testes and/or penis  2.. Fever OR leukocytosis + One or more of the following  - Costovertebral tenderness - Suprapubic tenderness - Gross hematuria  - Newly developed or aggravated frequency - Newly developed or aggravated urgency  - Newly developed or aggravated incontinence  3. Absence of fever or leukocytosis + Two or more of the following  - Suprapubic tenderness - Gross hematuria  - Newly developed or aggravated frequency - Newly developed or aggravated urgency  - Newly developed or aggravated incontinence |
| Existence of indwelling urinary catheter (Satisfaction with one or more of 1-4) | 1. Fever OR newly developed hypotension without evidence of infection in other sites  2. Acute change of mental status + leukocytosis  3. Costovertebral tenderness OR suprapubic tenderness  4. Gross pyuria OR acute pain/swelling or tenderness of the testes and/or penis |
| **Skin and soft tissue infection** | |
| Existence of four or more of the following | - Fever - Leukocytosis - Acute change of mental status  - Heating sense - Pain/Tenderness - Redness  - Serous or purulent discharge - Swelling |
| **Respiratory tract infection** | |
| Existence of findings with suspected pneumonia at chest radiography (Satisfaction with both 1 and 2) | 1. Existence of one or more of the following  - Newly developed or aggravated cough - Newly developed or aggravated sputum  - Oxygen saturation <94% (room air) or reduced >3% from baseline  - Pleuritic chest pain - Respiratory rate ≥25/min  - Newly developed or aggravated crackles on lung examination  2. Existence of one or more of the following: fever, leukocytosis, acute change of mental status |
| Absence of findings with suspected pneumonia at chest radiography (Satisfaction with both 1 and 2) | 1. Existence of two or more of the following:  - Newly developed or aggravated cough - Newly developed or aggravated sputum  - Oxygen saturation <94% (room air) or reduced >3% from baseline  - Pleuritic chest pain - Respiratory rate ≥25/min  - Newly developed or aggravated crackles on lung examination  2. Existence of one or more of the following: fever, leukocytosis, acute change of mental status |
| **Gastrointestinal tract infection** | |
| Satisfaction with one or more of 1-3 | 1. Diarrhea (three or more unformed stools in 24h period)  2. Vomiting (two or more in 24h period)  3. Causative microorganism was isolated from stool specimen + Existence of one or more of the following: diarrhea/vomiting/abdominal pain |
| *Clostridioides difficile* infection  (Satisfaction with both of 1-2) | 1. Diarrhea (three or more unformed stools in 24h period)  2. Positive result from *C. difficile* associated tests (CD culture, CD toxin ELISA/PCR, etc) OR pseudomembrane was found at endoscopy |
| **Ear, nose, throat infection** | |
| Ear infection: Existence of one or more of the following | - Diagnosed through examination by a physician  - Newly developed or aggravated discharge from one or both ears |
| Sinusitis | Diagnosed through examination by a physician |
| Group A Streptococcal acute pharyngotonsilitis (≥3 points) | (1 point) body temperature ≥38℃  (1 point) Absence of cough  (1 point) Tenderness on cervical lymph nodes  (1 point) Existence of swelling or discharge on tonsil  (1 point) Age 3-14 years  (0 point) Age 15-44 years  (-1 point) Age ≥44 years |
| **Other bloodstream infection** | |
| Satisfaction with all of 1-3 | 1. Organisms are isolated from blood cultures and thought not to be a contaminant  2. There are no evidence of other infections  3. Existence of one or more of the following: fever, hypotension, acute change of mental status |
| **Bone and joint infection** | |
| Existence of four or more of the following | - Fever - Leukocytosis - Acute change of mental status  - Heating sense - Pain/Tenderness - Redness  - Serous or purulent discharge - Swelling |
| **Infection with unknown origin** | |
| Satisfaction with all of 1-3 | 1. Fever  2. There are no evidence of other infections  3. Existence of one or more of the following: fever, hypotension, acute change of mental status |

Supplement 5. Antibiotics that were being used in each hospital

| A | B | C | D | E | F | G | H | I | J |
| --- | --- | --- | --- | --- | --- | --- | --- | --- | --- |
| Amoxicillin/Clavulanate  Cefaclor  Ceftazidime  Ceftriaxone  Ciprofloxacin  Clindamycin  Imipenem  Levofloxacin  Piperacillin/Tazobactam | Amikacin  Amoxicillin  Amoxicillin/Clavulanate  Cefaclor  Cefazolin  Cefotaxime  Cefradine  Ciprofloxacin  Clindamycin  Doxycycline  Gentamicin  Lincomycin | Amoxicillin  Cefaclor  Ceftriaxone  Ofloxacin | Amikacin  Ceftriaxone  Meropenem  Piperacillin/Tazobactam  Vancomycin | Amikacin  Amoxicillin  Amoxicillin/Clavulanate  Cefaclor  Cefradine  Ceftriaxone  Clindamycin  Doxycycline  Gentamicin  Imipenem  Levofloxacin  Metronidazole  Ofloxacin  Trimethoprim/Sulfamethoxazole | Amikacin  Amoxicillin/Clavulanate  Cefaclor  Cefazolin  Ceftazidime  Ceftriaxone  Ciprofloxacin  Colistin  Imipenem  Levofloxacin  Meropenem  Metronidazole  Piperacillin/Tazobactam  Trimethoprim/Sulfamethoxazole  Vancomycin | Amoxicillin/Clavulanate  Ampicillin/Sulbactam  Azithromycin  Cefaclor  Cefazolin  Cefdinir  Cefepime  Cefotaxime  Cefotetan  Cefpirome  Cefpodoxime  Ceftazidime  Ceftriaxone  Cefalexin  Cefazedone  Ciprofloxacin  Clarithromycin  Colistin  Flomoxef  Levofloxacin  Meropenem  Piperacillin/Tazobactam  Teicoplanin  Tetracycline | Amikacin  Amoxicillin  Amoxicillin/Clavulanate  Ampicillin/Sulbactam  Azithromycin  Benzathine penicillin  Cefaclor  Cefazolin  Cefixime  Cefotaxime  Cefprozil  Ceftazidime  Ceftezole  Ceftriaxone  Cefazedone  Clarithromycin  Clindamycin  Colistin  Doxycycline  Linezolid  Meropenem  Minocycline  Netilmicin  Piperacillin/Tazobactam  Ribostamycin  Roxithromycin  Teicoplanin  Vancomycin | Amikacin  Amoxicillin  Amoxicillin/Clavulanate  Ampicillin/Sulbactam  Azithromycin  Benzathine penicillin  Cefaclor  Cefadroxil  Cefazolin  Cefcapene  Cefditoren  Cefepime  Cefotaxime  Cefpodoxime  Ceftazidime  Ceftriaxone  Cefalexin  Cefazedone  Clarithromycin  Clindamycin  Colistin  Doripenem  Doxycycline  Ertapenem  Flomoxef  Gentamicin  Imipenem  Linezolid  Meropenem  Nafcillin  Piperacillin/Tazobactam  Teicoplanin  Tetracycline  Tigecycline  Trimethoprim/Sulfamethoxazole  Vancomycin | Amoxicillin  Amoxicillin/Clavulanate  Cefazolin  Cefixime  Cefradine  Ceftriaxone  Cefazedone  Ciprofloxacin  Ertapenem  Metronidazole  Netilmicin  Vancomycin |

Supplement 6. Antibiotic prescription pattern in each hospital

A. Calculated by DDD/1,000 patient-days

| Antibiotics | A | B | C | D | E | F | G | H | I | J |
| --- | --- | --- | --- | --- | --- | --- | --- | --- | --- | --- |
| 1st generation cephalosporin | - | 1.8 | - | - | 27.7 | 0.6 | 92.1 | 97.3 | 110.1 | 374.5 |
| 2nd generation cephalosporin | 8.7 | 6.1 | 6.8 | - | 12.8 | 19.0 | 131.3 | 59.7 | 83.4 | - |
| 3rd generation cephalosporin | 0.5 | 0.2 | 11.6 | 6.1 | 24.2 | 6.4 | 59.5 | 158.4 | 138.1 | 1.2 |
| 4th generation cephalosporin | - | - | - | - | - | - | 2.0 | - | 7.5 | - |
| Aminoglycoside | - | 3.0 | - | 1.1 | 16.1 | 0.0 | - | 14.8 | 2.7 | 0.9 |
| Beta-lactam/lactamase inhibitor | 37.0 | 15.4 | - | - | 10.1 | 40.1 | 31.6 | 68.0 | 58.6 | 0.4 |
| Beta-lactam/lactamase inhibitor (Anti-pseudomonal) | 34.2 | - | - | 27.4 | - | 1.6 | 25.3 | 27.6 | 18.5 | - |
| Carbapenem | 2.8 | - | - | 11.4 | 3.8 | 12.8 | 41.6 | 130.9 | 56.2 | 0.1 |
| Fluoroquinolone | 46.5 | 7.0 | 2.2 | - | 28.3 | 56.0 | 136.4 | - | - | 27.3 |
| Glycopeptide | - | - | - | 1.9 | - | 1.7 | 7.6 | 21.1 | 26.0 | 3.9 |
| Lincosamide | 1.7 | 5.2 | - | - | 2.0 | - | - | 0.5 | 5.4 | - |
| Macrolides | - | - | - | - | - | - | 138.4 | 26.5 | 11.5 | - |
| Metronidazole | - | - | - | - | 8.5 | 10.6 | - | - | - | 0.7 |
| Oxazolidinone | - | - | - | - | - | - | - | 0.2 | 0.2 | - |
| Penicillin | - | 5.3 | 9.3 | - | 20.6 | - | - | 3.4 | 4.6 | 1.2 |
| Polymyxin | - | - | - | - | - | 1.2 | 8.8 | 2.2 | 19.0 | - |
| Sulfonamide and trimethoprim | - | - | - | - | 14.5 | 9.2 | - | - | 1.3 | - |
| Tetracycline | - | 21.0 | - | - | 0.1 | - | 0.2 | 1.2 | 3.0 | - |
| Tigecycline | - | - | - | - | - | - | - | - | 0.4 | - |
| Total | 131.4 | 65.0 | 29.9 | 47.8 | 168.6 | 159.1 | 674.9 | 611.8 | 546.7 | 410.1 |

B. Calculated by DOT/1,000 patient-days

| Antibiotics | A | B | C | D | E | F | G | H | I | J |
| --- | --- | --- | --- | --- | --- | --- | --- | --- | --- | --- |
| 1st generation cephalosporin |  | 1.9 |  |  | 16.6 | 1.1 | 84.1 | 138.0 | 143.6 | 521.1 |
| 2nd generation cephalosporin | 13.4 | 6.2 | 9.1 |  | 16.9 | 27.5 | 185.0 | 42.5 | 87.4 |  |
| 3rd generation cephalosporin | 0.4 | 0.3 | 7.7 | 6.1 | 22.6 | 7.4 | 67.1 | 126.4 | 166.8 | 1.5 |
| 4th generation cephalosporin |  |  |  |  |  |  | 2.9 |  | 6.1 |  |
| Aminoglycoside |  | 6.3 |  | 2.2 | 34.0 | 0.0 |  | 27.3 | 4.7 | 1.1 |
| Beta-lactam/lactamase inhibitor | 38.3 | 11.5 |  |  | 21.3 | 57.6 | 36.9 | 73.3 | 70.9 | 1.1 |
| Beta-lactam/lactamase inhibitor (Anti-pseudomonal) | 34.0 |  |  | 35.2 |  | 3.2 | 34.0 | 23.9 | 22.0 |  |
| Carbapenem | 3.7 |  |  | 19.1 | 6.4 | 17.1 | 32.5 | 99.0 | 49.4 | 0.1 |
| Fluoroquinolone | 40.9 | 12.2 | 2.9 |  | 40.2 | 51.8 | 75.7 |  |  | 31.4 |
| Glycopeptide |  |  |  | 2.3 |  | 3.2 | 9.6 | 26.5 | 35.5 | 5.9 |
| Lincosamide | 4.6 | 11.4 |  |  | 2.1 |  |  | 0.7 | 5.9 |  |
| Macrolides |  |  |  |  |  |  | 54.6 | 18.0 | 8.5 |  |
| Metronidazole |  |  |  |  | 11.0 | 15.9 |  |  |  | 0.9 |
| Oxazolidinone |  |  |  |  |  |  |  | 0.2 | 0.2 |  |
| Penicillin |  | 3.6 | 19.1 |  | 19.1 |  |  | 0.4 | 2.0 | 0.8 |
| Polymyxin |  |  |  |  |  | 0.4 | 4.1 | 0.8 | 9.1 |  |
| Sulfonamide and trimethoprim |  |  |  |  | 19.7 | 19.9 |  |  | 2.1 |  |
| Tetracycline |  | 10.5 |  |  | 0.0 |  | 0.3 | 0.4 | 2.0 |  |
| Tigecycline |  |  |  |  |  |  |  |  | 0.4 |  |
| Total | 135.3 | 63.9 | 38.9 | 65.0 | 209.9 | 205.1 | 586.9 | 577.5 | 616.6 | 563.9 |

Supplement 7. Baseline characteristics for patients in the study for evaluation of appropriateness of antibiotic use, by hospitals

|  | A  (N=50) | B  (N=50) | C  (N=22) | D  (N=50) | E  (N=50) | F  (N=50) | G  (N=50) | H  (N=50) | I  (N=50) |
| --- | --- | --- | --- | --- | --- | --- | --- | --- | --- |
| Age, median (IQR) | 79.0  (72.0-87.3) | 80.0  (74.8-86.0) | 73.0  (61.0-85.5) | 80.0  (70.8-86.0) | 83.0  (76.8-87.0) | 81.0  (69.8-85.3) | 75.5  (57.8-85.0) | 77.5  (65.8-84.0) | 73.0  (59.0-84.3) |
| Female sex (%) | 20 (40.0) | 33 (66.0) | 10 (45.5) | 27 (54.0) | 24 (48.0) | 24 (48.0) | 24 (48.0) | 32 (64.0) | 22 (44.0) |
| Ward type (%) |  |  |  |  |  |  |  |  |  |
| General ward | 50 (100) | 50 (100) | 22 (100) | 50 (100) | 38 (76.0) | 50 (100) | 43 (86.0) | 32 (64.0) | 43 (86.0) |
| Intensive care unit | 0 | 0 | 0 | 0 | 12 (24.0) | 0 | 7 (14.0) | 18 (36.0) | 7 (14.0) |
| Classification of department (%) |  |  |  |  |  |  |  |  |  |
| Internal Medicine | 9 (18.0) | 0 | 0 | 13 (26.0) | 17/48 (35.4) | 29 (58.0) | 21 (42.0) | 46/49 (93.9) | 30 (60.0) |
| Medical department  (Excluding Internal Medicine) | 41 (82.0) | 41 (82.0) | 0 | 37 (74.0) | 22/48 (45.8) | 21 (42.0) | 1 (2.0) | 0 | 4 (8.0) |
| Surgical department | 0 | 9 (18.0) | 22 (100) | 0 | 9/48 (18.8) | 0 | 28 (56.0) | 3/49 (6.1) | 16 (32.0) |
| Availability of data about renal function at EMR (%) |  |  |  |  |  |  |  |  |  |
| Existence of result of CrCl | 28 (56.0) | 44 (88.0) | 0 | 0 | 0 | 0 | 0 | 0 | 50 (100) |
| Existence of result of eGFR | 0 | 44 (88.0) | 0 | 32 (64.0) | 0 | 0 | 14 (28.0) | 50 (100) | 50 (100) |
| Patients underwent renal replacement therapy (%) | 0 | 0 | 0 | 3 (6.0) | 0 | 21 (42.0) | 0 | 1 (2.0) | 5 (10.0) |
| Patients with cognitive disorder (%) | 47 (94.0) | 48 (96.0) | 8 (36.4) | 43 (86.0) | 45/48 (93.8) | 47 (94.0) | 9 (18.0) | 41 (82.0) | 22 (44.0) |
| Ambulation status |  |  |  |  |  |  |  |  |  |
| Ambulation, regardless of external support | 0 | 3 (6.0) | 4 (18.2) | 3 (6.0) | 3/48 (6.3) | 4 (8.0) | 35 (70.0) | 14 (28.0) | 20 (40.0) |
| Ambulation with wheelchair | 7 (14.0) | 7 (14.0) | 1 (4.5) | 9 (18.0) | 2/48 (4.2) | 19 (38.0) | 4 (8.0) | 13 (26.0) | 8 (16.0) |
| Bed-ridden status | 43 (86.0) | 40 (80.0) | 17 (77.3) | 38 (76.0) | 43/48 (89.6) | 27 (54.0) | 11 (22.0) | 23 (46.0) | 22 (44.0) |
| Availability of microbiological culture test |  |  |  |  |  |  |  |  |  |
| Existence of result of culture with blood sample | 0 | 0 | 0 | 19 (38.0) | 0 | 14 (28.0) | 19 (38.0) | 45/49 (91.8) | 38 (76.0) |
| Existence of result of culture with non-blood sample | 2 (4.0) | 0 | 0 | 21 (42.0) | 0 | 14 (28.0) | 20 (40.0) | 46 (92.0) | 37/48 (77.1) |

Abbreviations: IQR, interquartile range; EMR, electronic medical record; CrCl, creatinine clearance; eGFR, estimated glomerular filtration rate

Supplement 8. The characteristics of antibiotics subject to evaluation

|  | Long-term care hospitals  (N=384) | Acute care hospital  (N=185) | *P*-value | All hospitals  (N=569) |
| --- | --- | --- | --- | --- |
| Route of administration (%) |  |  | <0.001 |  |
| Parenteral | 245 (63.8) | 181 (97.8) |  | 426 (74.9) |
| Per oral | 139 (36.2) | 4 (2.2) |  | 143 (25.1) |
| Purpose of prescription^1^ (%) |  |  | <0.001 |  |
| Treatment of infectious diseases | 370/383 (96.6) | 155 (83.8) |  | 525/568 (92.4) |
| Genitourinary tract infection | 89/367 (24.3) | 33/159 (20.8) |  | 122/526 (23.2) |
| Skin and soft tissue infection | 28/367 (7.6) | 7/159 (4.4) |  | 35/526 (6.7) |
| Respiratory tract infection | 183/367 (49.9) | 74/159 (46.5) |  | 257/526 (48.9) |
| Gastrointestinal tract infection | 12/367 (3.3) | 37/159 (23.3) |  | 49/526 (9.3) |
| Eye infection | 0 | 0 |  | 0 |
| Ear, nose, throat infection | 13/367 (3.5) | 0 |  | 13/526 (2.5) |
| Other bloodstream infection | 1/367 (0.3) | 1/159 (0.6) |  | 2/526 (0.4) |
| Bone and joint infection | 4/367 (1.1) | 2/159 (1.3) |  | 6/526 (1.1) |
| Infection with unknown origin | 36/367 (9.8) | 5/159 (3.1) |  | 41/526 (7.8) |
| Other infections | 1/367 (0.3) | 0 |  | 1/526 (0.2) |
| Prophylaxis of surgical site infection | 3/383 (0.8) | 30/155 (16.2) |  | 33/568 (5.8) |
| Other or unknown reasons | 10/383 (2.6) | 0 |  | 10/568 (1.8) |
| Antibiotics subject to evaluation (%) |  |  |  |  |
| 1^st^ generation cephalosporin | 10 (2.6) | 19 (10.3) | <0.001 | 29 (5.1) |
| 2^nd^ generation cephalosporin | 21 (5.5) | 2 (1.1) | - | 23 (4.0) |
| 3^rd^ generation cephalosporin | 65 (16.9) | 39 (21.1) | - | 104 (18.3) |
| 4^th^ generation cephalosporin | 8 (2.1) | 1 (0.5) | - | 9 (1.6) |
| Aminoglycoside | 50 (13.0) | 2 (1.1) | - | 52 (9.1 |
| Beta-lactam/beta-lactamase inhibitor (Anti-pseudomonal) | 59 (15.4) | 40 (21.6) | - | 99 (17.4) |
| Beta-lactam/beta-lactamase inhibitor (Non-anti-pseudomonal) | 21 (5.5) | 0 | - | 21 (3.7) |
| Carbapenem | 22 (5.7) | 36 (19.5) | - | 58 (10.2) |
| Fluoroquinolone | 72 (18.8) | 32 (17.3) | - | 104 (18.3) |
| Glycopeptide | 3 (0.8) | 9 (4.9) | - | 12 (2.1) |
| Lincosamide | 7 (1.8) | 1 (0.5) | - | 8 (1.4) |
| Macrolide | 3 (0.8) | 0 | - | 3 (0.5) |
| Metronidazole | 13 (3.4) | 4 (2.2) | - | 17 (3.0) |
| Oxazolidinone | 0 | 0 | - | 0 |
| Penicillin | 20 (5.2) | 0 | - | 20 (3.5) |
| Polymyxin | 2 (0.5) | 0 | - | 2 (0.4) |
| Sulfonamide and trimethoprim | 4 (1.0) | 0 | - | 4 (0.7) |
| Tetracycline | 4 (1.0) | 0 | - | 4 (0.7) |
| Tigecycline | 0 | 0 | - | 0 |

^1^ One case was excluded due to incomplete data, six cases had multiple site infection (One case in long-term care hospital had a genitourinary tract infection and respiratory tract infection; one case in acute care hospital had a genitourinary tract infection and respiratory tract infection; four cases in acute care hospital had a respiratory tract infection and gastrointestinal tract infection)

Supplement 9. Antibiotics subject to evaluation and purpose of prescriptions, by hospitals

|  | A (N=74) | B (N=72) | C (N=27) | D (N=50) | E (N=107) | F (N=54) | G (N=60) | H (N=59) | I (N=66) |
| --- | --- | --- | --- | --- | --- | --- | --- | --- | --- |
| Antibiotics subject to evaluation (%) |  |  |  |  |  |  |  |  |  |
| 1^st^ generation cephalosporin | 0 | 0 | 3 (11.1) | 0 | 7 (6.5) | 0 | 14 (23.3) | 0 | 5 (7.6) |
| 2^nd^ generation cephalosporin | 0 | 1(1.4) | 9 (33.3) | 6 (12.0) | 0 | 5 (9.3) | 2 (3.3) | 0 | 0 |
| 3^rd^ generation cephalosporin | 12 (16.2) | 20 (27.8) | 1 (3.7) | 8 (16.0) | 22 (20.6) | 2 (3.7) | 15 (25.0) | 11 (18.6) | 13 (19.7) |
| 4^th^ generation cephalosporin | 0 | 8 (11.1) | 0 | 0 | 0 | 0 | 1 (1.7) | 0 | 0 |
| Aminoglycoside | 8 (10.8) | 2 (2.8) | 5 (18.5) | 0 | 35 (32.7) | 0 | 2 (3.3) | 0 | 0 |
| Beta-lactam/beta-lactamase inhibitor (Anti-pseudomonal) | 24 (32.4) | 0 | 0 | 10 (20.0) | 6 (5.6) | 19 (35.2) | 10 (16.7) | 14 (23.7) | 16 (24.2) |
| Beta-lactam/beta-lactamase inhibitor (Non-anti-pseudomonal) | 8 (10.8) | 9 (12.5) | 0 | 0 | 2 (1.9) | 2 (3.7) | 0 | 0 | 0 |
| Carbapenem | 3 (4.1) | 0 | 0 | 11 (22.0) | 1 (0.9) | 7 (13.0) | 6 (10.0) | 20 (33.9) | 10 (15.2) |
| Fluoroquinolone | 14 (18.9) | 22 (30.6) | 2 (7.4) | 11(22.0) | 8 (7.5) | 15 (27.8) | 9 (15.0) | 5 (8.5) | 18 (27.3) |
| Glycopeptide | 0 | 1 (1.4) | 0 | 0 | 0 | 2 (3.7) | 0 | 5 (8.5) | 4 (6.1) |
| Lincosamide | 1 (1.4) | 1 (1.4) | 0 | 0 | 5 (4.7) | 0 | 1 (1.7) | 0 | 0 |
| Macrolide | 0 | 0 | 0 | 3 (6.0) | 0 | 0 | 0 | 0 | 0 |
| Metronidazole | 3 (4.1) | 3 (4.2) | 0 | 0 | 6 (5.6) | 1 (1.9) | 0 | 4 (6.8) | 0 |
| Oxazolidinone | 0 | 0 | 0 | 0 | 0 | 0 | 0 | 0 | 0 |
| Penicillin | 0 | 1 (1.4) | 7 (25.9) | 1 (2.0) | 11 (10.3) | 0 | 0 | 0 | 0 |
| Polymyxin | 1 (1.4) | 0 | 0 | 0 | 0 | 1 (1.9) | 0 | 0 | 0 |
| Sulfonamide and trimethoprim | 0 | 0 | 0 | 0 | 4 (3.7) | 0 | 0 | 0 | 0 |
| Tetracycline | 0 | 4 (5.6) | 0 | 0 | 0 | 0 | 0 | 0 | 0 |
| Tigecycline | 0 | 0 | 0 | 0 | 0 | 0 | 0 | 0 | 0 |
| Route of administration (%) |  |  |  |  |  |  |  |  |  |
| Parenteral | 63 (85.1) | 33 (45.8) | 10 (37.0) | 22 (44.0) | 86 (80.4) | 31 (57.4) | 60 (100) | 58 (98.3) | 63 (95.5) |
| Per oral | 11 (14.9) | 39 (54.2) | 17 (63.0) | 28 (56.0) | 21 (19.6) | 23 (42.6) | 0 | 1 (1.7) | 3 (4.5) |
| Purpose of prescription (%) |  |  |  |  |  |  |  |  |  |
| Treatment of infectious diseases | 74 (100)^1^ | 70 (97.2)^2^ | 26/26 (100)^3^ | 50 (100)^4^ | 107 (100) | 43 (79.6) | 42 (70.0) | 59 (100)^5^ | 54 (81.8) |
| Genitourinary tract infection | 21/72 (29.2) | 19/68 (27.9) | 1/26 (3.8) | 21/51(41.2) | 24 (22.4) | 3 (7.0) | 4 (9.5) | 16/58 (27.6) | 13/59 (22.0) |
| Skin and soft tissue infection | 0 | 5/68 (7.4) | 8/26 (30.8) | 8/51 (15.7) | 3 (2.8) | 4 (9.3) | 3 (7.1) | 1/58 (1.7) | 3/59 (5.1) |
| Respiratory tract infection | 45/72 (62.5) | 24/68 (35.3) | 6/26 (23.1) | 17/51 (33.3) | 70 (65.4) | 21 (48.8) | 19 (45.2) | 27/58 (46.6) | 28/59 (47.5) |
| Gastrointestinal tract infection | 1/72 (1.4) | 5/68 (7.4) | 1/26 (3.8) | 0 | 2 (1.9) | 3 (7.0) | 12 (28.6) | 12/58 (20.7) | 13/59 (22.0) |
| Eye infection | 0 | 0 | 0 | 0 | 0 | 0 | 0 | 0 | 0 |
| Ear, nose, throat infection | 0 | 4/68 (5.9) | 1/26 (3.8) | 2/51 (3.9) | 2 (1.9) | 4 (9.3) | 0 | 0 | 0 |
| Other bloodstream infection | 0 | 0 | 0 | 1/51 (2.0) | 0 | 0 | 0 | 0 | 1/59 (1.7) |
| Bone infection | 1/72 (1.4) | 0 | 0 | 0 | 2 (1.9) | 1 (2.3) | 1 (2.4) | 0 | 1/59(1.7) |
| Infection with unknown origin | 4/72 (5.6) | 10/68 (14.7) | 9/26 (34.6) | 2/51 (3.9) | 4 (3.7) | 7 (16.3) | 3 (7.1) | 2/58 (3.4) | 0 |
| Other infections | 0 | 1/68 (1.5) | 0 | 0 | 0 | 0 | 0 | 0 | 0 |
| Prophylaxis of surgical site infection | 0 | 2 (2.8) | 0 | 0 | 0 | 1 (1.9) | 18 (30.0) | 0 | 12 (18.2) |
| Others | 0 | 0 | 0 | 0 | 0 | 10 (18.5) | 0 | 0 | 0 |

^1,2^ Two cases were not able to be classified because the data was insufficient.

^3^ One case was excluded because the data was insufficient,

^4^ One patient had two infectious diseases simultaneously.

^5^ One case was not able to be classified because the data was insufficient.

^6^ Five patient had two infectious diseases simultaneously.

Supplement 10. Appropriateness of antibiotic prescriptions, by hospitals

|  | A (N=74) | B (N=72) | C (N=27) | D (N=50) | E (N=107) | F (N=54) | G (N=60) | H (N=59) | I (N=66) |
| --- | --- | --- | --- | --- | --- | --- | --- | --- | --- |
| Route of administration (%) |  |  |  |  |  |  |  |  |  |
| Appropriate | 74 (100) | 72 (100) | 26 (96.3) | 43 (86.0) | 107 (100) | 52 (96.3) | 60 (100) | 59 (100) | 63 (95.5) |
| Inappropriate | 0 | 0 | 1 (3.7) | 7 (14.0) | 0 | 2 (3.7) | 0 | 0 | 3 (4.5) |
| Dose (%) |  |  |  |  |  |  |  |  |  |
| Optimal | 30 (40.5) | 30 (41.7) | 0 | 23 (46.0) | 2 (1.9) | 27 (50.0) | 31 (34.0) | 57 (96.6) | 51 (77.3) |
| Suboptimal: excessively high dose | 10 (13.5) | 5 (6.9) | 1 (3.7) | 2 (4.0) | 1 (0.9) | 13 (24.1) | 2 (3.3) | 0 | 0 |
| Inappropriate: excessively low dose | 6 (8.1) | 35 (48.6) | 0 | 13 (26.0) | 6 (5.6) | 2 (3.7) | 10 (16.7) | 2 (3.4) | 13 (19.7) |
| N/A | 28 (37.8) | 2 (2.8) | 26 (96.3) | 12 (24.0) | 98 (91.6) | 12 (22.2) | 17 (28.3) | 0 | 2 |
| Antibiotic choice (%) |  |  |  |  |  |  |  |  |  |
| Antibiotics for the treatment of infectious diseases |  |  |  |  |  |  |  |  |  |
| Appropriateness of diagnosis | 71/74 (95.9) | 23/70 (32.9) | 11/26 (42.3)^1^ | 42/50 (84.0) | 104/107 (97.2) | 31/43 (72.1) | 20/42 (47.6) | 47/59 (79.7) | 52/54 (96.3) |
| Appropriateness of antibiotic prescription |  |  |  |  |  |  |  |  |  |
| Optimal | 57/74 (77.0) | 10/70 (14.3) | 8/26 (30.8)^1^ | 19/50 (38.0) | 33/107 (30.8) | 19/43 (44.2) | 12/42 (28.6) | 33/59 (55.9) | 37/54 (68.5) |
| Suboptimal | 0 | 2/70 (2.9) | 1/26 (3.8)^1^ | 16/50 (32.0) | 34/107 (31.8) | 7/43 (16.3) | 8/42 (19.0) | 6/59 (10.2) | 2/54 (3.7) |
| Inappropriate | 11/74 (14.9) | 48/70 (68.6) | 9/26 (34.6)^1^ | 15/50 (30.0) | 36/107 (33.6) | 10/43 (23.3) | 21/42 (50.0) | 11/59 (18.6) | 12/54 (22.2) |
| N/A | 6/74 (8.1) | 10/70 (14.3) | 8/26 (30.8)^1^ | 0 | 4/107 (3.7) | 7/43 (16.3) | 1/42 (2.4) | 9/59 (15.3) | 3/54 (5.6) |
| Antibiotics for the prophylaxis of surgical site infection |  |  |  |  |  |  |  |  |  |
| Appropriate | - | 0 | - | - | - | 1/1 (100) | 12/18 (66.7) | - | 5/12 (41.7) |
| Inappropriate | - | 2/2 (100) | - | - | - | 0 | 6/18 (33.3) | - | 7/12 (58.3) |
| Antibiotics for other or unknown reasons |  |  |  |  |  |  |  |  |  |
| Appropriate | - | - | - | - | - | 0 | - | - | - |
| Inappropriate | - | - | - | - | - | 10/10 (100) | - | - | - |
| Appropriateness of antibiotic prescription, by each antibiotic (%) |  |  |  |  |  |  |  |  |  |
| Optimal | 22 (29.7) | 5 (6.9) | 0 | 10 (20.0) | 2 (1.9) | 10 (18.5) | 9 (15.0) | 31 (52.5) | 36 (54.5) |
| Suboptimal | 9 (12.2) | 3 (4.2) | 0 | 10 (20.0) | 0 | 7 (13.0) | 5 (8.3) | 6 (10.2) | 1 (1.5) |
| Inappropriate | 13 (17.6) | 52 (72.2) | 0 | 18 (36.0) | 7 (6.5) | 19 (35.2) | 28 (46.7) | 13 (22.0) | 24 (36.4) |
| N/A | 30 (40.5) | 12 (16.7) | 27 (100) | 12 (24.0) | 98 (91.6) | 18 (33.3) | 18 (30.0) | 9 (15.3) | 5 (7.6) |
| Appropriateness of antibiotic prescription, by each patient (%) |  |  |  |  |  |  |  |  |  |
| Optimal | 12/50 (24.0) | 2/50 (4.0) | 0 | 10/50 (20.0) | 0 | 5/50 (10.0) | 8/50 (16.0) | 25/50 (50.0) | 24/50 (48.0) |
| Suboptimal: one or more antibiotics were suboptimal | 7/50 (14.0) | 2/50 (4.0) | 0 | 10/50 (20.0) | 0 | 7/50 (14.0) | 3/50 (6.0) | 5/50 (10.0) | 1/50 (2.0) |
| Suboptimal: unnecessary combination therapy | 0 | 0 | 0 | 0 | 0 | 1/50 (2.0) | 0 | 0 | 4/50 (8.0) |
| Inappropriate | 11/50 (22.0) | 36/50 (72.0) | 1/50 (2.0) | 18/50 (36.0) | 1/50 (2.0) | 19/50 (38.0) | 22/50 (44.0) | 12/50 (24.0) | 16/50 (32.0) |
| N/A | 20/50 (40.0) | 10/50 (20.0) | 49/50 (98.0) | 12/50 (24.0) | 49/50 (98.0) | 18/50 (36.0) | 17/50 (34.0) | 8/50 (16.0) | 5/50 (10.0) |

^1^ One case was excluded because the data was insufficient,

Supplement 11. Inappropriately prescribed antibiotics

| Antibiotics | N=174 |
| --- | --- |
| 1^st^ generation cephalosporin | 0 |
| 2^nd^ generation cephalosporin | 7 (4.0) |
| 3^rd^ generation cephalosporin | 34 (19.5) |
| 4^th^ generation cephalosporin | 5 (2.9) |
| Aminoglycoside | 5 (2.9) |
| Beta-lactam/beta-lactamase inhibitor (Anti-pseudomonal) | 20 (11.5) |
| Beta-lactam/beta-lactamase inhibitor (Non-anti-pseudomonal) | 14 (8.0) |
| Carbapenem | 11 (6.3) |
| Fluoroquinolone | 50 (28.7) |
| Glycopeptide | 6 (3.4) |
| Lincosamide | 3 (1.7) |
| Macrolide | 3 (1.7) |
| Metronidazole | 11 (6.3) |
| Penicillin | 2 (1.1) |
| Polymyxin | 0 |
| Sulfonamide and trimethoprim | 0 |
| Tetracycline | 3 (1.7) |

Supplement 12. Appropriateness of antibiotic prescriptions by infectious diseases

|  | Optimal | Suboptimal | Inappropriate | N/A | Total |
| --- | --- | --- | --- | --- | --- |
| Total hospitals, n=514 (%) |  |  |  |  |  |
| Genitourinary tract infection | 22 (18.3) | 16 (13.3) | 35 (29.2) | 47 (39.2) | 120 |
| Skin and soft tissue infection | 4 (11.4) | 2 (5.7) | 11 (31.4) | 18 (51.4) | 35 |
| Respiratory tract infection | 60 (23.9) | 15 (6.0) | 71 (28.3) | 105 (41.8) | 251 |
| Gastrointestinal tract infection | 20 (44.4) | 7 (15.6) | 12 (26.7) | 6 (13.3) | 45 |
| Ear, nose, throat infection | 1 (7.7) | 1 (7.7) | 5 (38.5) | 6 (46.2) | 13 |
| Other bloodstream infection | 0 | 0 | 1 (50.0) | 1 (50.0) | 2 |
| Bone and joint infection | 1 (16.7) | 0 | 0 | 5 (83.3) | 6 |
| Infection with unknown origin | 2 (4.8) | 0 | 13 (31.0) | 27 (64.3) | 42 |
| Long-term care hospitals, n=365 (%) |  |  |  |  |  |
| Genitourinary tract infection | 14 (15.9) | 9 (10.2) | 22 (25.0) | 43 (48.9) | 88 |
| Skin and soft tissue infection | 3 (10.7) | 2 (7.1) | 9 (32.1) | 14 (50.0) | 28 |
| Respiratory tract infection | 27 (14.8) | 12 (6.6) | 47 (25.8) | 96 (52.7) | 182 |
| Gastrointestinal tract infection | 3 (25.0) | 5 (41.7) | 2 (16.7) | 2 (16.7) | 12 |
| Ear, nose, throat infection | 1 (7.7) | 1 (7.7) | 5 (38.5) | 6 (46.2) | 13 |
| Other bloodstream infection | 0 | 0 | 0 | 1 (100) | 1 |
| Bone and joint infection | 0 | 0 | 0 | 4 (100) | 4 |
| Infection with unknown origin | 1 (2.7) | 0 | 11(29.7) | 25 (67.6) | 37 |
| Acute care hospitals, n=149 (%) |  |  |  |  |  |
| Genitourinary tract infection | 8 (25.0) | 7 (21.9) | 13 (40.6) | 4 (12.5) | 32 |
| Skin and soft tissue infection | 1 (14.3) | 0 | 2 (28.6) | 4 (57.1) | 7 |
| Respiratory tract infection | 33 (47.8) | 3 (4.3) | 24 (34.8) | 9 (13.0) | 69 |
| Gastrointestinal tract infection | 17 (51.5) | 2 (6.1) | 10 (30.3) | 4 (12.1) | 33 |
| Other bloodstream infection | 0 | 0 | 1 (100) | 0 | 1 |
| Bone and joint infection | 1 (50.0) | 0 | 0 | 1 (50.0) | 2 |
| Infection with unknown origin | 1 (20.0) | 0 | 2 (40.0) | 2 (40.0) | 5 |

Note: Antibiotic prescriptions in patients who had two infectious diseases simultaneously were excluded (n=6).
